# Neural correlate of reduced respiratory chemosensitivity during chronic epilepsy

**DOI:** 10.3389/fncel.2023.1288600

**Published:** 2023-12-20

**Authors:** Amol M. Bhandare, Nicholas Dale

**Affiliations:** School of Life Sciences, University of Warwick, Coventry, United Kingdom

**Keywords:** epilepsy, seizure, sudden unexpected death in epilepsy (SUDEP), respiratory chemosensitivity, *in vivo* imaging, retrotrapezoid nucleus (RTN), rostral ventrolateral medullary (RVLM)

## Abstract

While central autonomic, cardiac, and/or respiratory dysfunction underlies sudden unexpected death in epilepsy (SUDEP), the specific neural mechanisms that lead to SUDEP remain to be determined. In this study, we took advantage of single-cell neuronal Ca^2+^ imaging and intrahippocampal kainic acid (KA)-induced chronic epilepsy in mice to investigate progressive changes in key cardiorespiratory brainstem circuits during chronic epilepsy. Weeks after induction of status epilepticus (SE), when mice were experiencing recurrent spontaneous seizures (chronic epilepsy), we observed that the adaptive ventilatory responses to hypercapnia were reduced for 5 weeks after SE induction with its partial recovery at week 7. These changes were paralleled by alterations in the chemosensory responses of neurons in the retrotrapezoid nucleus (RTN). Neurons that displayed adapting responses to hypercapnia were less prevalent and exhibited smaller responses over weeks 3–5, whereas neurons that displayed graded responses to hypercapnia became more prevalent by week 7. Over the same period, chemosensory responses of the presympathetic rostral ventrolateral medullary (RVLM) neurons showed no change. Mice with chronic epilepsy showed enhanced sensitivity to seizures, which invade the RTN and possibly put the chemosensory circuits at further risk of impairment. Our findings establish a dysfunctional breathing phenotype with its RTN neuronal correlate in mice with chronic epilepsy and suggest that the assessment of respiratory chemosensitivity may have the potential for identifying people at risk of SUDEP.

## 1 Introduction

Every year, the mortality rates due to sudden unexpected death in epilepsy (SUDEP) in people with epilepsy and people with drug-resistant epilepsy are 0.4-2 per 1,000 persons and 4-9 per 1,000 persons, respectively (Tomson et al., 2008; Ryvlin et al., 2013). There is currently no treatment or biomarker test available to identify people who are at a higher risk of SUDEP. Although previous studies showed that SUDEP mainly occurs in people with drug-resistant and generalized tonic–clonic seizures (Tomson et al., 2008; Ryvlin et al., 2013), it is still unclear why these seizures are more fatal in people with a history of seizures (Shorvon and Tomson, 2011). In addition, it is still unknown whether the incidence of SUDEP is higher in people with frequent seizures because each seizure has a fixed and finite chance of precipitating death or because there are cumulative changes in central cardiorespiratory circuitry resulting from a history of seizures that progressively increase the risk of death. There is some evidence that cumulative autonomic cardiorespiratory changes due to repeated seizures might be a potential cause of SUDEP (Patodia et al., 2022).

Central autonomic cardiorespiratory brainstem circuits generate the rhythms for breathing and the sympathetic and parasympathetic activity that controls the heart (Spyer and Gourine, 2009; Guyenet, 2014). Evidence from epilepsy monitoring units (So et al., 2000; Tomson et al., 2008; Ryvlin et al., 2013; Sivathamboo et al., 2020) and findings from *in vivo* rodent studies (Aiba and Noebels, 2015; Jefferys et al., 2019) imply that apnoea (either centrally mediated or obstructive) and bradycardia occurring immediately after seizures lead to SUDEP. Numerous studies have proposed factors that may contribute to SUDEP, including parasympathetic hyperactivity (Kalume et al., 2013), deficiency of Kv1.1 potassium channels (Glasscock et al., 2010; Moore et al., 2014; Aiba and Noebels, 2015), reduced heart rate variability (Surges et al., 2009), cardiac arrhythmia (Naggar et al., 2014; Bhandare et al., 2015; Stewart, 2018), hypoxemia (Bateman et al., 2010), hypercapnia (Seyal et al., 2010), pulmonary oedema (So et al., 2000), airway obstruction (Stewart et al., 2017; Jefferys et al., 2019; Irizarry et al., 2020), amygdala seizures (Park et al., 2020), the role of neurotransmitters serotonin (Faingold et al., 2011; Buchanan et al., 2014) and adenosine (Faingold et al., 2016; Shen et al., 2022) in seizures, and loss of brainstem volume (Mueller et al., 2014) or glial cells (Patodia et al., 2019). The origins of SUDEP are likely to lie in the dysfunction of key brainstem circuits (So et al., 2000; Ryvlin et al., 2013; Mueller et al., 2014). In genetic mouse models of epilepsy, seizure-induced spreading depolarisation (recorded in the dorsal medulla) silences the brainstem respiratory neuronal network, and this interference has been proposed to cause SUDEP (Aiba and Noebels, 2015). While this finding indicates disruption or silencing of key cardiorespiratory outputs from the brainstem as a cause of SUDEP, it does not provide insights into the specific neuronal circuitry and mechanisms involved.

A recent study has shown reduced ventilatory responses to hypercapnic challenges up to 30 days after pilocarpine-induced status epilepticus (SE) in rats (Maia et al., 2020). Additionally, lower hypercapnic ventilatory responses in people with epilepsy have been correlated with higher postictal transcutaneous CO_2_ increase following generalized convulsive seizures (Sainju et al., 2019). As the drive to breathe is critically dependent on PCO_2_, reductions in sensitivity to PCO_2_/pH of central chemosensory neurons might plausibly contribute to central apnoea and ultimately SUDEP. These chemosensory neurons are located at the ventral surface of the medulla and regulate breathing to maintain systemic PCO_2_ within physiological limits (Spyer and Gourine, 2009; Guyenet, 2014). Cardiovascular function is controlled by excitatory rostral ventrolateral medullary (RVLM) neurons in the brainstem (Spyer and Gourine, 2009; Guyenet, 2014). To address whether progressive changes in epilepsy alter the neuronal firing patterns and chemosensitivity in these critical brainstem networks, we used intravital microscopy to record these brainstem nuclei in freely behaving mice (Bhandare et al., 2022).

By using genetically encoded Ca^2+^ indicators and gradient refractive index (GRIN) optic fibers, to allow the direct visualization of dynamic cellular activity of deep brain cardiorespiratory neurons in awake mice at single-cell resolution, we have recorded how seizures alter the activity and responses of chemosensitive respiratory RTN and cardiovascular RVLM neurons. We found that mice with chronic epilepsy (a state characterized by unprovoked recurrent seizures), compared to their pre-epileptic state, have reduced baseline respiratory activity (tidal volume, V_T_) for 5 weeks after induction of epilepsy. In addition, the adaptive breathing changes to hypercapnia were substantially weakened for 5 weeks following SE and showed partial recovery at week 7. By simultaneously recording neuronal activity from the same mice before and following the establishment of SE for several weeks, we have documented parallel changes in the chemosensory responses of RTN neurons that could explain the observed changes in the chemosensory control of breathing. Although these parallel changes do not demonstrate a mechanism of SUDEP, we propose that the reduced chemosensitivity of RTN neurons, by lessening the baseline drive to breathe and reducing adaptive ventilatory responses to hypercapnia, could be a contributing mechanism of SUDEP.

## 2 Materials and methods

Experiments were performed in accordance with the European Commission Directive 2010/63/EU (European Convention for the Protection of Vertebrate Animals used for Experimental and Other Scientific Purposes) and the United Kingdom Home Office (Scientific Procedures) Act (1986) with project approval from the University of Warwick's AWERB (PP1674884).

### 2.1 Viral handling

We used AAV-9-syn-GCaMP6s vector with synapsin promoter (pGP-AAV-syn-GCaMP6s-WPRE.4.641 at a titer of 1 × 10^13^ GC·ml^−1^, Addgene, Watertown, MA, USA), and therefore, it transduced neurons showing higher tropism for the AAV 2/9 subtype and did not transduce non-neuronal cells. The AAV uses the synapsin promoter. The virus was aliquoted and stored at −80°C. On the day of injection, it was removed and stored at 4°C, loaded into graduated glass pipettes (Drummond Scientific Company, Broomall, PA, USA), and placed into an electrode holder for pressure injection.

### 2.2 Viral transfection of RTN and RVLM neurons

**Figure 1:**
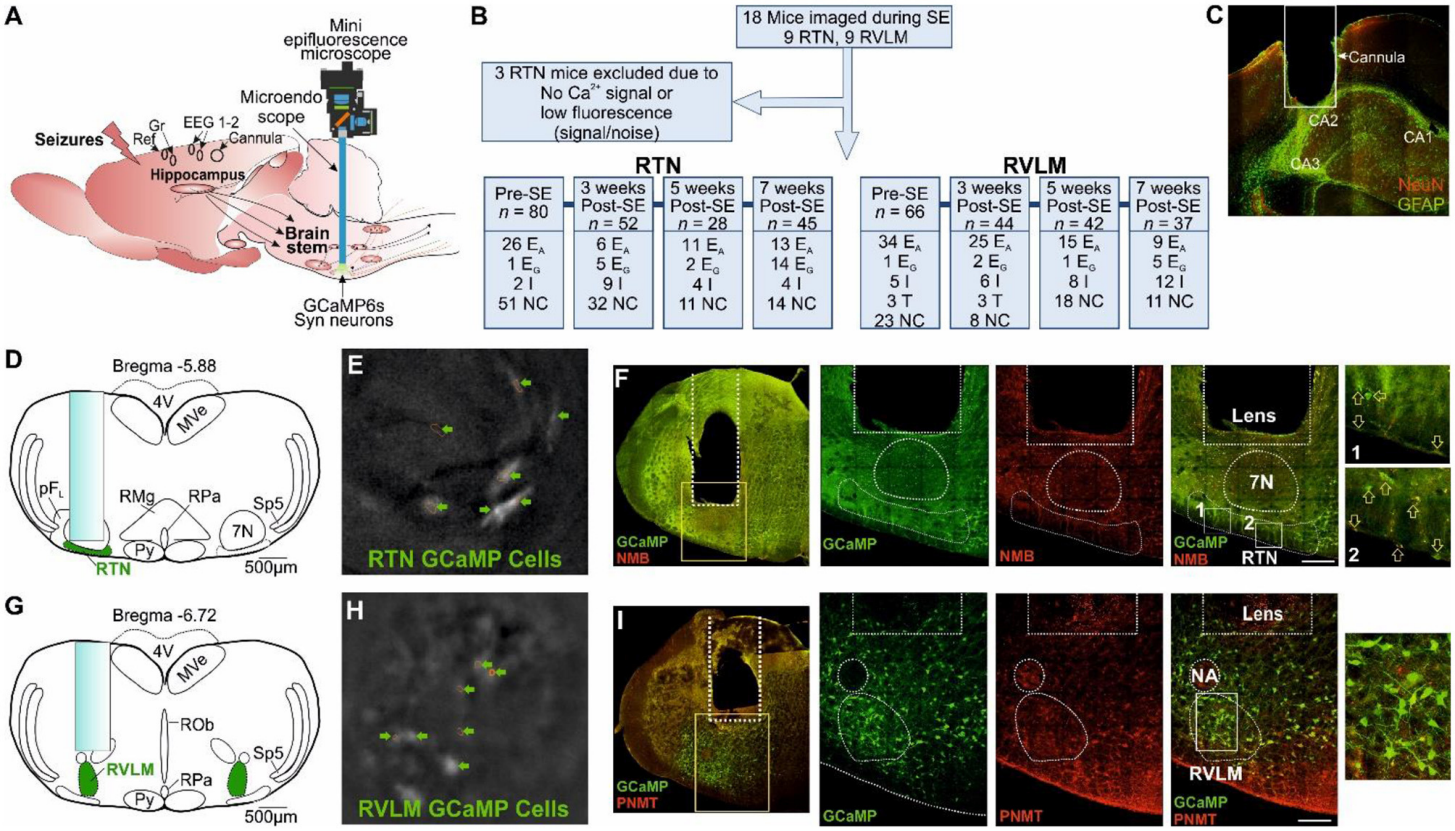
Microendoscopic experimental approach to target specific brainstem nuclei. **(A)** Representation of GRIN lens (microendoscope), baseplate, and mini epifluorescence camera placement for the recording of brainstem nuclei with a hippocampal cannula for seizure induction and EEG (recording, reference and ground) electrodes. **(B)** A CONSORT flow diagram for inclusion/exclusion of experiments and cell classification. Definitions of the range of firing patterns in response to hypercapnia are described in the text. “*n”* represents the number of neurons at a specific time point. **(C)** Micrograph of the hippocampal cannula placement for injection of KA. **(D)** AAV9-Syn-GCaMP6s injection into the RTN with the lens position. **(E)** GCaMP6s fluorescence of transduced RTN neurons in freely behaving mice. **(F)** Micrograph of lens placement and viral transduction of neurons (green) relative to the facial nucleus (7N) and NMB+ RTN neurons (red). Arrow represents the NMB+ GCaMP6+ neurons. **(G)** AAV9-Syn-GCaMP6s injection into the RVLM with the lens position. **(H)** GCaMP6s fluorescence of transduced RVLM neurons in freely behaving mice. **(I)** Micrograph of lens placement and viral transduction of neurons (green) and PNMT+ RVLM neurons (red). Scale bar in **(F, I)** represents 200 μm; E_A_, adapting; NC, non-coding; I, inhibited; E_G_, graded; T, tonic: 7N, facial motor nucleus; Py, pyramidal tract; MVe, medial vestibular nucleus; sp5, spinal trigeminal nucleus: RTN, retrotrapezoid nucleus; RMg, raphe magnus; RPa, raphe pallidus; ROb, raphe obscurus; pFL, parafacial lateral region; RVLM, rostral ventrolateral medulla; NA, nucleus ambiguus; NeuN, neuronal nuclei; GFAP, glial fibrillary acidic protein; NMB, neuromedin B; PNMT, phenylethanolamine N-methyltransferase.

Adult male C57BL/6J mice (8–10 weeks old and 20–30 g) were randomly selected from the cage and assigned to the injection group. Mice were anesthetized with isoflurane (4%; Piramal Healthcare Ltd., Mumbai, India) in pure oxygen (4 L·min^−1^). Adequate anesthesia was maintained with 0.5–2% isoflurane in pure oxygen (1 L·min^−1^) throughout the surgery. Mice received a pre-surgical subcutaneous injection of atropine (120 μg·kg^−1^; Westward Pharmaceutical Co., Eatontown, NJ, USA) and meloxicam (2 mg·kg^−1^; Norbrook Inc., Lenexa, KS, USA). Mice were placed in a prone position in a digital stereotaxic apparatus (Kopf Instruments, Tujunga, CA, USA) on a heating pad (TCAT 2-LV: Physitemp, Clifton, NJ, USA), and body temperature was maintained at a minimum value of 33°C via a thermocouple. The head was leveled at Bregma and 2 mm caudal to Bregma, and graduated glass pipettes containing the virus were placed stereotaxically into either the RTN or RVLM (Figures 1D, G). The RTN was defined as the area ventral to the caudal half of the facial nucleus, which bound medially and laterally by the edges of the facial nucleus (coordinates with a 9° injection arm angle: −1.0 mm lateral and −5.6 mm caudal from Bregma and −5.2 to −5.5 mm ventral from the surface of the cerebellum; Figure 1D). The RVLM is defined as a triangular area ventral to the nucleus ambiguus (NA), lateral to the inferior olive (IO) or pyramids (Py), and medial to the spinal trigeminal sensory nucleus (Sp5) (coordinates with a 0° injection arm angle: 1.3 mm lateral and −5.7 mm caudal from Bregma, and −5.15 mm ventral from the surface of the cerebellum; Figure 1G). The virus solution was pressure injected (<300 nL) unilaterally. Pipettes were left in place for 3–5 min to prevent the backflow of the virus solution up the pipette track. Postoperatively, mice received intraperitoneal (IP) injections of buprenorphine (100 μg·kg^−1^; Reckitt Benckiser, Slough, UK). Mice were allowed 2 weeks for recovery and viral expression, with food and water *ad libitum*.

### 2.3 Implantation of EEG electrodes, hippocampal cannula, and GRIN lens

Mice expressing GCaMP6 were anesthetized with isoflurane, given pre-surgical drugs, placed into a stereotax, and the head was leveled as described above. Four holes were drilled for the implantation of two EEG recordings, a ground and a reference electrode (Figure 1A). EEG electrodes were PFA-coated silver wire of diameter: 0.254 mm (Bilaney Consultants Ltd., UK) (coordinates with a 0° injection arm angle: recording electrodes- ±1.5 mm lateral and 1.0 mm rostral from Bregma, ground and reference electrodes- ±2.5 mm lateral and −1.5 mm caudal from Bregma). Silver wires were implanted and secured in position with SuperBond™ (Prestige Dental, Bradford, UK). EEG wires were passed through the pedestal (Bilaney Consultants Ltd., UK), and the pedestal was implanted and secured in place over the head with SuperBond™. Another two holes were drilled each for the implantation of unilateral hippocampal cannula (Figures 1A, C, coordinates with a 0° injection arm angle: 1.8 mm lateral and −2.0 mm caudal from Bregma, and −1.6 mm ventral from the surface of the dura) and GRIN lens (coordinates as above). A stainless steel cannula, 26 gauge and 10 mm long, was implanted in the hippocampus and secured in place with SuperBond™. To widen the lens path whilst producing the least amount of deformation of tissue, a graduated approach was taken; first, a glass pipette was inserted down the GRIN lens path to a depth of 500 μm above where the lens would be terminated and left in place for 3 min; this procedure was then repeated with a blunted hypodermic needle. The GRIN lens (600 μm diameter, 7.3 mm length; Inscopix, Palo Alto, CA, USA) was then slowly inserted at a rate of 100 μm·min^−1^ to a depth of ~1,300 μm above the target site and then lowered at a rate of 50 μm·min^−1^ to a depth of approximately 300 μm above the RTN or RVLM target region (coordinates as above with 300 μm above the target site). The lens was then secured in place with SuperBond™. Postoperatively, mice received buprenorphine and were allowed 2 weeks for recovery, with food and water *ad libitum*.

### 2.4 Baseplate installation

Mice expressing GCaMP6 and implanted with EEG electrodes, hippocampal cannula, and GRIN lens were anesthetized with isoflurane, given pre-surgical drugs, and placed into a stereotax as described above. To hold the miniaturized microscope during recordings, a baseplate was positioned over the lens and adjusted until the cells under the GRIN lens were in focus. The baseplate was then secured with SuperBond™ and coated in black dental cement (Vertex Dental, Soesterberg, the Netherlands) to stop the interference of the recording from ambient light. Mice were allowed 1 week for recovery, with food and water *ad libitum*.

### 2.5 Induction of status epilepticus (SE) with EEG recording and Ca^2+^ imaging in freely moving mice

All mice were trained with a dummy camera and habituated to plethysmography and the open field Plexiglass chamber before imaging. Instrumented mice were anesthetized with isoflurane and placed into a stereotax as described above. Mice EEG electrodes were connected to wires, and a miniature microscope with an integrated 475 nm LED (Inscopix, Palo Alto, CA, USA) was secured into the baseplate. SE was induced in mice with a total of 0.3 μg (in 50 nl volume) of unilateral kainic acid (KA) injection through the hippocampal cannula with Hamilton Neuros syringes (Model 75 RN, Essex Scientific Laboratory Supplies Ltd., UK). Mice were taken off the anesthesia and placed into an open field chamber. The EEG recording and Ca^2+^ imaging were started ~6 min after KA injection. Mice were scored every minute for their behavioral seizures using the Racine scale (Racine, 1972; Lüttjohann et al., 2009): 1—sudden behavioral arrest and/or motionless staring; 2—facial jerking with muzzle or muzzle and eye (stiffened and arched tail); 3—neck jerks (or head bobbing and partial body clonus); 4—clonic seizure in a sitting position; 5—convulsions including clonic and/or tonic–clonic seizures while lying on the belly and/or pure tonic seizures; and 6—convulsions including clonic and/or tonic–clonic seizures while lying on the side and/or wild jumping. At 60 min or recording 1–2 of class 5/6 scale seizure following injection of KA (whichever happened earlier), mice were anesthetized with isoflurane, and SE was terminated with an intraperitoneal injection of ketamine (50 mg/kg) and diazepam (20 mg/kg).

During SE, EEG activity was recorded, amplified, and filtered using the NeuroLog system (Digitimer, Welwyn Garden City, UK) connected to a 1401 interface and was acquired on a computer using *Spike2* software (Cambridge Electronic Design, Cambridge, UK). The EEG activity raw data were DC removed. The power in the “gamma” frequency range (25–45 Hz) was analyzed, as previously shown (Gurbanova et al., 2008; Bhandare et al., 2016). A power spectrum analysis of EEG (AUC, V^2^) was done from 2-min blocks taken every 4 min apart after the start of recording. Video data were recorded with *Spike2* software and were synchronized with the EEG activity (Supplementary videos 1, 2). GCaMP6 fluorescence was visualized during SE through the GRIN lens, using nVista 2 HD acquisition software (Inscopix, Palo Alto, CA, USA). Calcium fluorescence was optimized for each experiment so that the histogram range was approximately 150–600, with average recording parameters set at 10 frames/s with the LED light's power set to 10–20 mW and a digital gain of 1.0–4.0. A TTL pulse was used to synchronize the calcium signaling to the behavioral data and EEG trace. All images were processed using Inscopix data processing software (Inscopix, Palo Alto, CA, USA). GCaMP6 movies were run through a preprocessing algorithm (with spatial and temporal downsampling), crop, spatial filter algorithm (0.005–0.5 Hz), motion correction, and cell identification through Constrained Nonnegative Matrix Factorization for microEndoscopic data (CNMF-E) analysis to generate the identified cell sets. Cell sets were imported into *Spike2* software for processing. All Ca^2+^ traces from specific time points were averaged and analyzed for the area under the curve (AUC) between the start of 3% hypercapnia and 180 s after it.

### 2.6 Plethysmography

Mice were placed into a custom-made 0.5-L plethysmography chamber, with an airflow rate of 1 L·min^−1^. The plethysmography chamber was heated to 31°C (thermoneutral for C57/BL6 mice). CO_2_ concentrations were sampled via a Hitech Instruments (Luton, UK) GIR250 Dual Sensor Gas analyser or ML206 gas analyser (AD Instruments, Sydney, Australia) connected to the inflow immediately before entering the chamber. The analyser had a delay of ~15–20 s to read out the digital output of the gas mixture. Pressure transducer signals and CO_2_ measurements were amplified and filtered using the NeuroLog system (Digitimer, Welwyn Garden City, UK) connected to a 1401 interface and acquired on a computer using *Spike2* software (Cambridge Electronic Design, Cambridge, UK). Video data were recorded with *Spike2* software and were synchronized with the breathing trace. Airflow measurements were used to calculate tidal volume (V_T_: signal trough at the end of expiration subtracted from the peak signal during inspiration, converted to mL following calibration) and respiratory frequency (*f* R: breaths per minute). Minute ventilation (V_E_) was calculated as V_T_ x *f* R.

### 2.7 Hypercapnia in freely behaving mice

Mice were tested for hypercapnic challenge before and 3, 5, and 7 weeks after induction of SE. Instrumented mice were allowed approximately 30 min to acclimate to the plethysmograph. The LED was activated through a TTL pulse synchronized with the *Spike2* recording, and 3 min of baseline recordings was taken (gas mixture: 0% CO_2_ 21% O_2_ 79% N_2_). The mice were then exposed to 3 min epochs of the hypercapnic gas mixture at different concentrations of CO_2_ (3% and 6% in 21% O_2_ balanced N_2_). Following exposure to the hypercapnic gas mixtures, CO_2_ levels were reduced back to 0%, and calcium signals were recorded for a further recovery period of 4 min.

Hypercapnic breathing responses were tested in the following groups of mice:

1. The RTN epileptic (SE-induced) group (*n* = 9).
2. The RVLM epileptic (SE-induced) group (*n* = 9, except at week 3, where *n* = 6 as it was not possible to record from three mice due to pandemic restrictions).
3. Control group of mice (*n* = 6): these were sham-operated to match with SE induction groups.

Each mouse was considered to be an independent statistical replicate.

### 2.8 Seizure threshold in mice with chronic epilepsy

After recording 3-, 5-, and 7-week post-SE hypercapnia responses and during the chronic phase of epilepsy, 8 weeks after induction of SE mice were tested for seizure threshold. Instrumented mice were anesthetized with isoflurane, placed into a stereotax, and connected to EEG electrodes and a miniature microscope as described above. Mice were injected through intrahippocampal cannula with a 3- or 6-fold lower dose of KA (0.1 μg, *n* = 4 and 0.05 μg, *n* = 10 of KA/mouse) compared to SE. Mice were taken off the anesthesia and placed into an open field chamber, and EEG recording and Ca^2+^ imaging were started as stated above. Mice were recorded until the first EEG seizure burst was recorded (Supplementary videos 3–4), and seizures were terminated as above.

### 2.9 Ca^2+^ imaging, data analysis, and inclusion/exclusion criteria for cells in the study

Using methods that we have recently developed (Bhandare et al., 2022), neurons of the RTN and RVLM were transduced with an AAV to drive expression of Ca^2+^ reporter GCaMP6 under the control of the *hSyn* promoter and to allow the assessment of their activity via a head-mounted mini-microscope (Figure 1A). We prepared 18 mice (9 with an injection of GCaMP6 into the RTN and 9 with injection into the RVLM, Figure 1B) and assessed their responses to hypercapnia before and weeks after induction of SE (during the chronic phase of epilepsy; 3, 5, and 7 weeks post-SE). After the exclusion of three RTN-injected mice (Figure 1B) for insufficient fluorescence signals, we analyzed the remaining mice for their neuronal responses to hypercapnia before induction of SE and at 3, 5, and 7 weeks following SE induction with intrahippocampal KA injection (Figure 1C, Supplementary Figures 1–2).

As the mice were unrestrained and able to move freely during the recordings to visualize physical movements during Ca^2+^ analysis, high-definition videos of mice were synchronized to the simultaneous plethysmograph and Inscopix Ca^2+^ imaging recordings in Inscopix Data Processing Software and Spike2. Our recordings were performed at a single wavelength (Figure 1A). Therefore, it was essential to verify that any signals result from changes in Ca^2+^ rather than from movement artifacts.

Detailed procedures to assess and compensate for movement artifacts have been presented in previously studies (Bhandare et al., 2022). In brief, if there was too much uncompensated motion artifact so that we could not clearly analyse Ca^2+^ signals, we excluded these recordings from quantitative analysis. GCaMP6 signals were only accepted for categorization if the following criteria were met: (1) The features of the cell (e.g., soma, large processes) could be clearly seen; (2) they occurred in the absence of movement of the mouse or were unaffected by mouse movement; (3) fluorescence changed relative to the background; and (4) the focal plane had remained constant as shown by other non-fluorescent landmarks (e.g., blood vessels). To assess the role of movement, we examined the GCaMP6 fluorescence in conjunction with the mouse's body movement. We included the cells that that did not evoke noticeable change in GCaMP6 fluorescence with the body movements and were characterized by a fast rise and exponential decay in the absence of any movement and during movement.

Following assessment for the inclusion/exclusion criteria for the imaging of both RTN and RVLM mice (Figure 1A), data from 15 mice comprising recordings from 146 (pre-SE), 96 (week 3 post-SE), 60 (week 5 post-SE), and 82 (week 7 post-SE) cells were included for the analysis (Figure 1B).

We found different neuronal patterns as we have shown previously (Bhandare et al., 2022), and these patterns are as follows:

*Inhibited (I)*: Displayed spontaneous Ca^2+^ activity at rest, which was greatly reduced during both 3% and 6% inspired CO_2_ and could exhibit rebound activity following the end of the hypercapnic episode.

*Excited adapting (E*_*A*_*)*: Silent or with low-level activity at rest and showed the greatest Ca^2+^ activity in response to a change in 3% inspired CO_2_. Following an initial burst of activity, they were either silent or displayed low-level activity throughout the remainder of the hypercapnic episode. These cells often exhibited rebound activity following the end of the hypercapnic episode.

*Excited graded (E*_*G*_*):* Silent or with low-level activity at rest and displayed an increase in Ca^2+^ activity at 3% inspired CO_2_ with a further increase in activity at 6% that returned to baseline upon the removal of the stimulus.

*Tonic (T):* Displaying spontaneous Ca^2+^ activity throughout the recording that was unaffected by the hypercapnic episode.

*Non-coding (NC):* Displayed low frequency or sporadic Ca^2+^ activity that had no discernible relationship to the hypercapnic stimulus or any respiratory event.

We transduced the RTN in nine mice and the RVLM in a further nine mice. After the exclusion criteria, six of the RTN mice and all of the RVLM mice were retained for the imaging analysis (Figure 1B). Recordings from individual neurons were considered independent statistical replicates.

### 2.10 Statistical analysis

Statistical analysis was performed in GraphPad Prism software (version 9.1.0). Statistical significance for changes in breathing was determined using two-way repeated measure ANOVA followed by *t*-tests with Tukey's correction and shown as a violin plot. Statistical analysis of the changes in RTN and RVLM neuronal adapting hypercapnic response, baseline activity, and changes during SE and subthreshold KA-induced seizures was performed via the Brown–Forsythe and Welch ANOVA with Dunnett's T3 correction. This alternative test was used as the data were not normally distributed and the variance differed between groups in the comparison, thus invalidating the use of a conventional ANOVA. The seizure delay and duration time were analyzed using a one-way ANOVA followed by Dunnett's correction. The results are shown as a violin plot with superimposed data points. The Racine seizure scores between the two groups (violin plot with superimposed data points) and a number of adapting vs. non-adapting neuronal responses (donut charts) were compared with the chi-squared test and corrected with the false discovery rate procedure for multiple comparisons (Curran-Everett, 2000). Comparisons were done between pre- and post-treatment (SE or control groups). A *p*-value of ≤ 0.05 was considered statistically significant. ^****^p ≤ 0.0001, ^***^p ≤ 0.001, ^**^p ≤ 0.01, and ^*^p ≤ 0.05. The results are presented as mean ± SEM throughout the text.

### 2.11 Immunohistochemistry

Mice were humanely killed by pentobarbital overdose (>100 mg·kg^−1^) and transcardially perfused with paraformaldehyde solution (4% PFA; Sigma-Aldrich, St Louis, MO, USA). The head was removed and postfixed in PFA (4°C) for 3 days to preserve the lens tract. The brains were removed and postfixed in PFA (4°C) overnight. Brainstems or hippocampus were serially sectioned at 50–70 μm. Free-floating sections were incubated for 1 h in a blocking solution (PBS containing 0.1% Triton X-100 and 5% BSA). Primary antibodies (rabbit anti-Neuromedin-B [NMB; 1:1,000; SAB1301059; Sigma-Aldrich, St Louis, MO, USA], rabbit anti-phenylethanolamine N-methyltransferase [PNMT; 1:500; 204179-T36-SIB; Stratech, Ely, UK] antibody), (chicken anti-GFAP [GFAP; 1:500; ab4674; Abcam PLC, Cambridge, UK], or mouse anti-NeuN [NeuN; 1:500; ab104224; Abcam PLC, Cambridge, UK]) were added, and the tissue was incubated overnight at room temperature.

Slices were washed in PBS (6 × 5 min), and then, the secondary antibodies were added: Either donkey anti-rabbit Alexa Fluor 680 (1:250; Jackson Laboratory, Bar Harbor, ME, USA), donkey anti-rabbit Alexa Fluor 594 (1:250; Jackson Laboratory, Bar Harbor, ME, USA), donkey anti-mouse Alexa Fluor 680 (1:250; Jackson Laboratory, Bar Harbor, ME, USA) antibody, or donkey anti-chicken Alexa Fluor 594 (1:250; Jackson Laboratory, Bar Harbor, ME, USA) antibody tissue was then incubated for 2–4 h at room temperature. The tissue was washed in PBS (6 × 5 min). Slices were mounted, coverslipped, and examined using a Zeiss 880 confocal microscope with ZEN acquisition software (Zeiss, Oberkochen, Germany).

## 3 Results

### 3.1 Seizures spread into the central autonomic cardiorespiratory network

We were unable to record neuronal activity during spontaneous seizures, and all the neuronal responses shown here are from evoked seizures and during response to hypercapnia in mice, which is because the low frequency of spontaneous seizures and their unpredictable occurrence make it difficult to record the neuronal activity during these seizures. Ca^2+^ imaging cannot be performed continuously due to the bleaching of GCaMP6. Thus, it is not possible to systematically record neuronal activity during these rare seizures. Neurons recorded in the RTN were targeted with the GRIN lens (Figure 1D), imaged in freely behaving mice (Figure 1E), and identified by their position relative to the facial (seventh) nucleus and co-staining for neuromedin B (NMB) (Figure 1F). Neurons in the RVLM were targeted with the GRIN lens (Figure 1G), imaged in freely behaving mice (Figure 1H), and identified by their position relative to the nucleus ambiguus and immunoreactivity for phenylethanolamine N-methyltransferase (PNMT) (Figure 1I).

**Figure 2:**
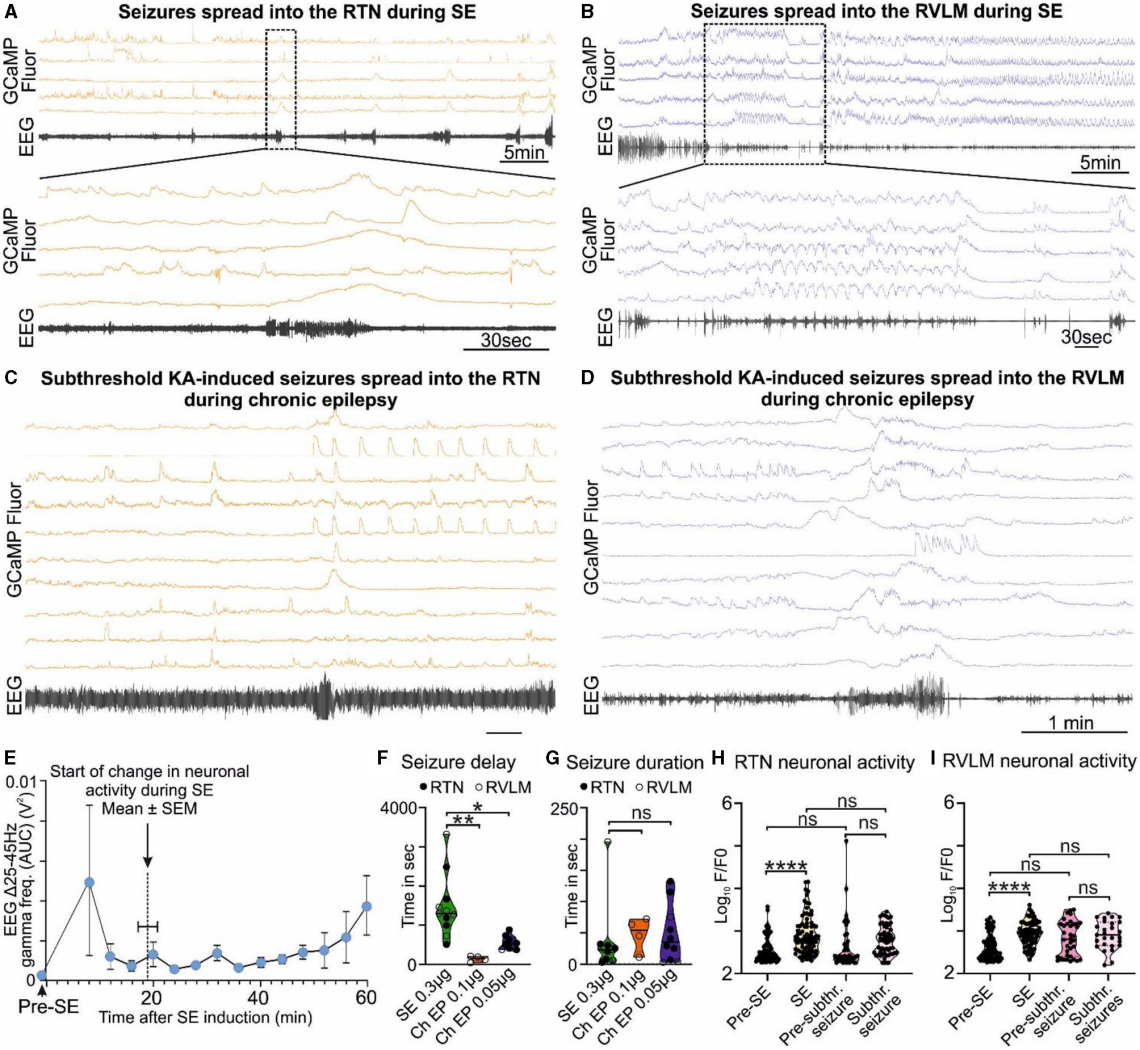
KA-induced seizures alter the activity of brainstem cardiorespiratory neurons. **(A)** KA-induced seizures during status epilepticus (SE) spread into the RTN nuclei (representative trace; *n* = 9). The dotted square shows changes in the activity of RTN neurons in freely behaving mice time matched with EEG seizure and expanded under it. Seizure activity after intrahippocampal injection of KA time matched with the activity of RTN neurons (orange). **(B)** KA-induced seizures during status epilepticus spread into the RVLM nuclei (representative trace; *n* = 9). The dotted square shows changes in the activity of RVLM neurons in freely behaving mice time matched with EEG seizure and expanded under it. Seizure activity after intrahippocampal injection of KA time matched with the activity of RVLM neurons (blue). The effect of 6- and 3-fold lower doses of intrahippocampal KA (compared with the SE) on **(C)** the RTN and **(D)** the RVLM neuronal activities, respectively, with reduced seizure induction delay time. **(E)** Start of seizure activity, i.e., changes in amplitude of gamma-range frequency (25–45 Hz) before induction of SE (pre-SE) and after intrahippocampal injection of KA (*n* = 8). Increase in the gamma range frequency (25–45 Hz) in EEG is a characteristic property of KA-induced seizures (Gurbanova et al., 2008; Bhandare et al., 2016). The seizure start time is compared with the time of first visual aberrant cardiorespiratory neuronal activity calculated from respective Ca^2+^ traces from mice recorded for RTN and RVLM (*n* = 14). The start of a change in neuronal activity is represented as a dotted line. During chronic epilepsy in mice, seizure delay **(F)** and duration time **(G)** with 3- and 6-fold lower doses of intrahippocampal KA (against SE) are compared with times at SE. *n* = 11 at SE (0.3 μg of intrahippocampal KA), and *n* = 4 and 10 (0.1 and 0.05 μg of intrahippocampal KA, respectively) during chronic epilepsy. The average change in the activity of all RTN **(H)** and RVLM **(I)** neurons during induction of SE and subthreshold KA-induced seizures in comparison with their pre-level (baseline) activities, i.e., before induction of SE and before induction of subthreshold KA-induced seizures during chronic epilepsy (at week 7 after induction of SE). Data in **(F–I)** are median and quartile and shown as continuous and dotted lines, respectively. *P*-values derived from one-way ANOVA with multiple comparisons and corrected with Dunnett's multiple comparisons test for **(F, G)** and Brown–Forsythe test for **(H, I)**. F* (DFn, DFd) are 2.796 (Ryvlin et al., 2013; Park et al., 2020) for **(F)**, 0.3038 (Ryvlin et al., 2013; Park et al., 2020) for **(G)**, 12.68 (3.000, 150.6) for **(H)**, and 39.70 (3.000, 106.7) for **(I)**. The significant *P*-values are *p < 0.05, **p < 0.01, and ****p < 0.0001. subthr., subthreshold.

During SE-induced seizures (recorded via EEG electrodes), large amplitude Ca^2+^ signals could be observed in neurons of both the RTN (Figure 2A, Supplementary Figure 3A, Supplementary movie 1) and RVLM (Figure 2B, Supplementary Figure 4A, Supplementary movie 2) groups. This aberrant neuronal activity commenced approximately 18.9 ± 1.5 min after injection of KA (Figure 2E; there was ~6 min delay between intrahippocampal injection and the start of recording) and lasted throughout the period of seizures until this was terminated by ketamine and diazepam injection. We could not record the EEG before the injection of intrahippocampal KA as the position of the hippocampal cannula was very close to the EEG headmount, and this would have necessitated anesthetizing the mice twice (for connection of the EEG electrodes pre-SE and during intrahippocampal KA injection). Due to this technical reason, we did not record the EEG prior to intrahippocampal KA injection; however, as shown in previous studies (Cavalheiro et al., 1982; Gurbanova et al., 2008; Bhandare et al., 2016), we found that the seizures had already started at 6 min after KA injection [increase in the power of the gamma-range frequency (25–45 Hz)] compared to pre-seizure EEG activity (EEG activity during pre-SE hypercapnic response; Figure 2E). During SE induction through intrahippocampal injection of KA (0.3 μg/mouse), we recorded the first seizure (EEG burst) with EEG electrodes at 1,481.8 ± 240.5 s (mean ± SEM) after KA injection (Figure 2F), and the seizure lasted for 33.7 ± 16.4 s (Figure 2G).

### 3.2 Increased sensitivity and reduced latency to seizure induction with aberrant brainstem neuronal activity in mice with chronic epilepsy

At 7.9 ± 0.2 weeks after induction of SE (i.e., during the chronic phase of epilepsy), the seizures induced by the lower doses of KA (0.1 μg of KA/mouse, *n* = 4, and 0.05 μg of KA/mouse, *n* = 10) were able to invade the RTN (Figure 2C, Supplementary movie 4, 0.05 μg of KA) and RVLM (Figure 2D, Supplementary movie 3, 0.1 μg of KA) groups and cause aberrant neuronal activity within these nuclei (Supplementary Figures 3B, B). We found that the time for the induction of the first seizure/visual EEG burst was significantly reduced compared to the onset of seizures during SE [142.0 ± 37.7 s for 0.1 μg KA, *p* = 0.0008, Figure 2F, Supplementary movie 3 (RVLM imaging) and 552.8 ± 53.5 s for 0.05 μg KA, p = 0.0016, Figure 2G, Supplementary movie 4 (RTN imaging)], but there was no change in the duration of the resulting seizures (47.8 ± 13.5 s for 0.1 μg KA, p = 0.8460, and 48.6 ± 14.5 s for 0.05 μg KA, p = 0.7190; Figures 2F–G) as compared with SE.

We compared the baseline activity of all RTN and RVLM neurons before induction of SE with activity during SE and at week 7 post-SE (before subthreshold KA-induced seizures) with the activity during subthreshold KA-induced seizures in chronic epilepsy (Figures 2H–I). The activity of all neurons in both nuclei was significantly increased at SE compared to pre-SE (*p* < 0.0001 for both RTN and RVLM) but did not change at subthreshold KA-induced seizures compared to their pre-levels (*p* = 0.4512 and 0.9163 for the RTN and RVLM, respectively). Most importantly, between the RTN and RVLM groups, there was no difference in the activity levels at SE and lower doses of KA-induced seizures compared to pre-SE and subthreshold KA-induced seizures, respectively (Figures 2H–I). The baseline activity in both nuclei, when recorded for all neurons, showed no difference between the pre-SE level and the level recorded at 7 weeks post-SE (*p* = 0.9716 and 0.0662 for the RTN and RVLM groups, respectively; Figures 2H–I).

Our findings show that the activity of the cardiorespiratory brainstem neuronal network, although far from the site of seizure origin, can still be altered by the subthreshold (3- and 6-fold lower) dose of KA-induced seizures in the hippocampus during the chronic phase of epilepsy. This change plausibly suggests a mechanism for the cardiorespiratory failure during SUDEP: aberrant neuronal activity in the RTN and RVLM during and immediately after a seizure. The equal magnitude of brainstem neuronal responses to SE and subthreshold dose of KA during chronic epilepsy (*p* = 0.3152 and 0.9992 for the RTN and RVLM groups, respectively) suggests an increased sensitivity and susceptibility of brainstem cardiorespiratory neuronal network to seizures during chronic epilepsy that could plausibly arise from continued invasion of spontaneous seizures into the brainstem nuclei (Figures 2H–I).

### 3.3 Development of a compromised breathing phenotype in mice with chronic epilepsy

**Figure 3:**
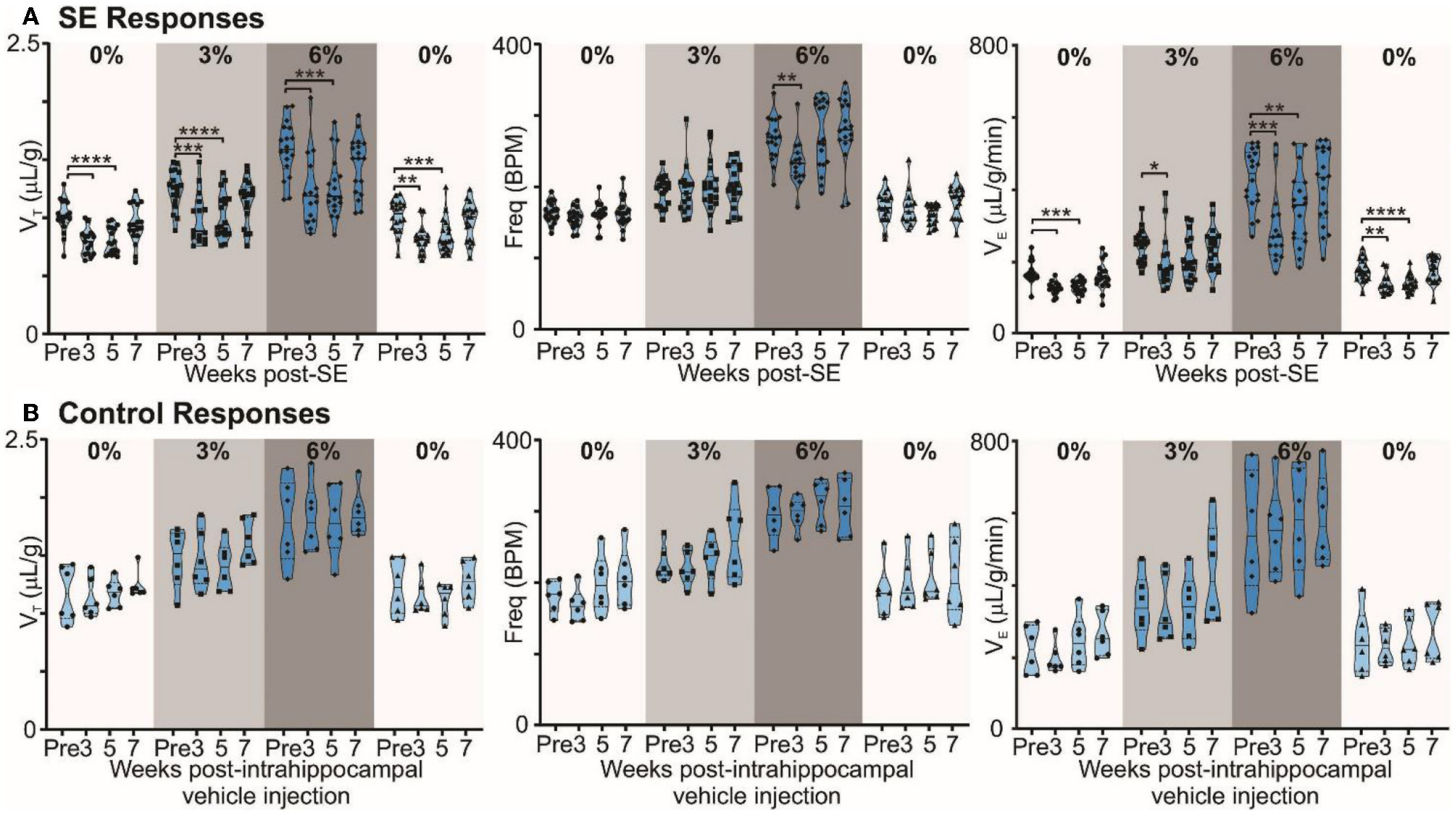
Chronic epilepsy induces a breathing phenotype in mice. **(A)** Changes in tidal (V_T_), frequency (f_R_), and minute ventilation (V_E_) in response to 3% and 6% CO_2_ before and 3, 5, and 7 weeks after induction of SE in freely behaving mice (*n* = 18). Median and quartile are shown with continuous and dotted lines, respectively. **(B)** Changes in tidal (V_T_), frequency (f_R_), and minute ventilation (V_E_) in response to 3% and 6% CO_2_ before and 3, 5, and 7 weeks after injection of intrahippocampal PBS in freely behaving mice (control; *n* = 6). Data are median and quartile and are shown as continuous and dotted lines, respectively. *P*-values derived from two-way repeated measure (mixed effects) ANOVA with Tukey's multiple comparisons are ^*^p < 0.05, ^**^p < 0.001, ^***^p < 0.001, and ^****^p < 0.0001.

The RTN is an important nucleus that contributes to the chemosensory control of breathing. By contrast, the RVLM projects to the sympathetic and parasympathetic preganglionic neurons and controls cardiovascular parameters such as heart rate and blood pressure. RVLM neurons also exhibit chemosensitivity (Koganezawa and Paton, 2014). As seizures invade these nuclei, we expected that there might be altered chemosensory control during the development of chronic epileptic states following induction of SE. We, therefore, examined how responses to hypercapnia in mice subjected to intrahippocampal KA injection or saline injection (control, surgical intervention but no SE induction) changed with time (Figure 3). We evaluated hypercapnic neuronal and breathing responses from week 3 post-SE in mice that were already experiencing spontaneous seizures and compared them with pre-SE responses. As the mice were already experiencing spontaneous seizure at week 3 post-SE, this means that our post-SE neuronal and breathing responses were outside the classical latent period (Levesque and Avoli, 2013; Lévesque et al., 2016; Rusina et al., 2021).

In mice with chronic epilepsy, the baseline tidal volume (V_T_) during breathing of room air was significantly lowered when assessed 3 and 5 weeks after induction of SE (*p* < 0.0001; Figure 3A). The increases in V_T_ observed during mild (3% CO_2_) and moderate (6% CO_2_) hypercapnia were also depressed compared to their values pre-SE (*p* = 0.0004 and <0.0001 for 3% CO_2_ at 3 and 5 weeks post-SE, respectively, and p = 0.0003 and 0.0001 for 6% CO_2_ at 3 and 5 weeks post-SE, respectively; Figure 3A). At week 7, epileptic mice showed partial recovery of reduced V_T_ responses to hypercapnia (*p* = 0.3644 and 0.3155 at 3% and 6% CO_2_, respectively, compared to pre-SE levels; Figure 3A). Interestingly, the respiratory frequency was not altered by induction of SE or during the establishment of chronic epilepsy. As might be expected, the changes in minute ventilation (V_E_) reflected the changes apparent in V_T_ (Figure 3A). The effect of induction of epilepsy on baseline breathing and breathing during hypercapnia can also be seen in Supplementary Figure 5, which replots data from Figure 3 as V_T_ vs. inspired CO_2_. The inset plot in Supplementary Figure 5 shows that, at 6% CO_2_ challenge, there is a significant reduction in chemosensitivity at weeks 3 (23.3%) and 5 (19.6%) compared to the pre-SE state.

By contrast, in control mice, the ventilatory responses to hypercapnia were maintained in control mice over the entire study period (Figure 3B). V_T_ in baseline (room air breathing) became stronger at 7 weeks post-intrahippocampal saline injection (*p* = 0.83; Figure 3B). The lung inspiratory capacity of mice increases with age with maximum change happening between 3 and 12 months (Huang et al., 1985; Schulte et al., 2019). An increasing baseline breathing trend observed in the control mice is attributable to this maturation (Figure 3B). The age of control and epileptic mice at the pre-SE/PBS recording was between 90 and 170 days, and at 7 weeks post-SE/PBS recording, it was between 145 and 230 days. There was no significant age difference between the two groups at pre- (*p* = 0.66) and 7 weeks post-SE (*p* = 0.21) recordings (unpaired *t*-test with Welch's correction). Following induction of SE, the normal maturational increase in baseline breathing was not observed (compare Figures 3A, B), which could be due to the effect of SE or the development of chronic epilepsy in these mice. Overall, our data show that the alteration in baseline breathing and the sensitivity of breathing to different levels of CO_2_ evident in the epileptic mice are due to the induction of epilepsy rather than the surgical intervention.

### 3.4 Attenuated responses to hypercapnia during chronic epilepsy by adapting RTN chemosensitive neurons

**Figure 4:**
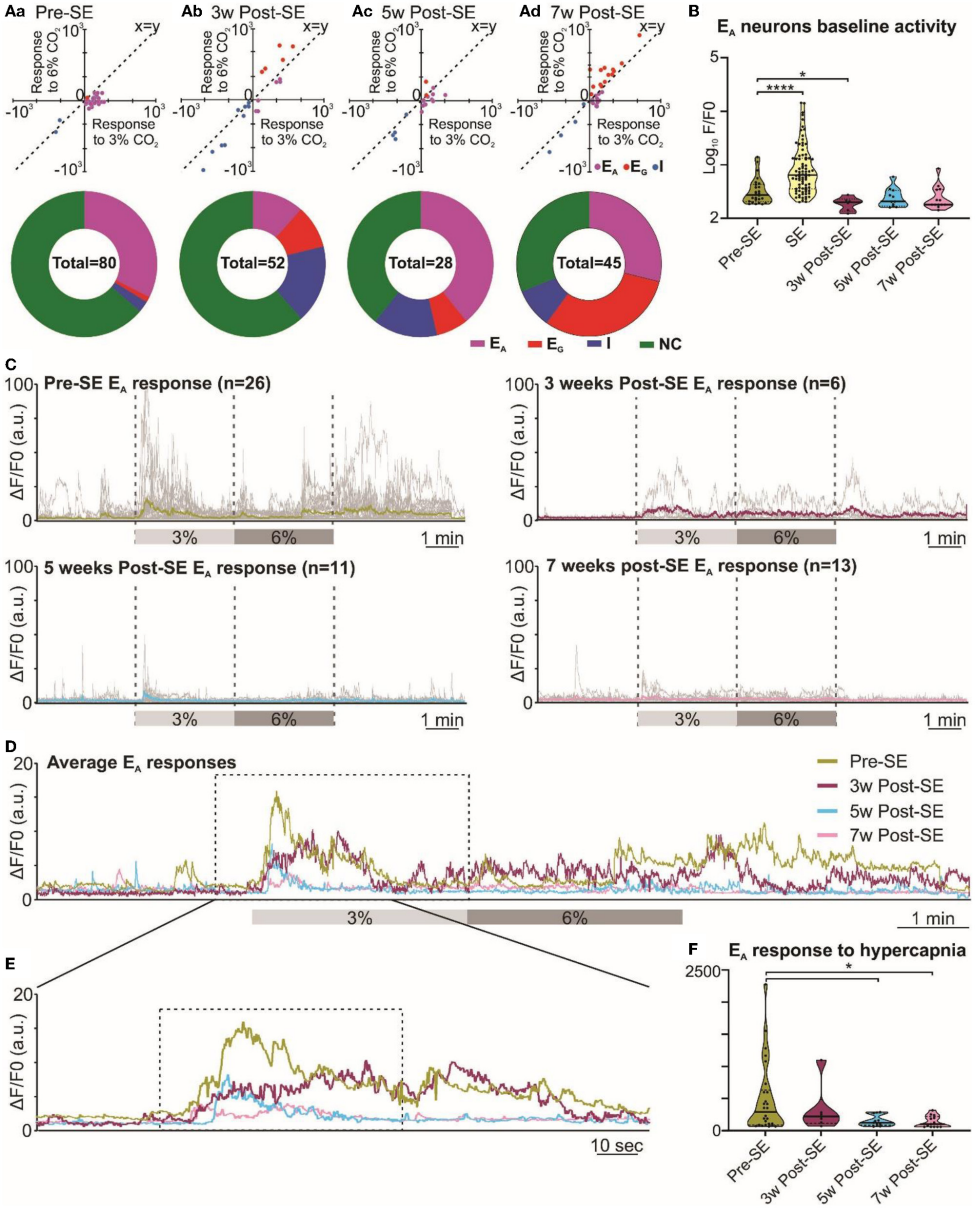
Altered RTN chemosensitive neuronal response to hypercapnia during chronic epilepsy. **(A)** Two-component analysis of neuronal categorization of RTN cells at pre-SE (a) and 3 (b), 5 (c), and 7 weeks (d) after induction of SE. The change in Ca^2+^ signal (measured as the area under the curve) from baseline (room air) elicited by 6% CO_2_ plotted against the Ca^2+^ response elicited by 3% CO_2_. The line of identity (x = y) is shown. E_A_ neurons fall below this line, whereas E_G_ neurons fall above this line and I neurons are predominant in the negative quadrant of the graph. The donut charts represent the proportion of different types of neuronal responses to hypercapnia in the RTN at pre-SE (a) and 3 (b), 5 (c), and 7 weeks (d) after induction of SE. **(B)** Baseline activities of RTN Ad neurons at pre-SE and 3, 5, and 7 weeks after induction of SE and compared with neuronal responses during SE. **(C)** RTN neurons individual and average adapting responses to hypercapnia pre-SE and 3, 5, and 7 weeks after induction of epilepsy in freely behaving mice. n is the number of neurons recorded from six mice. In 7-week post-SE panel, for technical reasons, the recording of recovery from hypercapnia is absent. **(D)** The RTN neurons average adapting responses compared between pre-SE and 3, 5, and 7 weeks after induction of SE. The dotted square shows changes in adapting response of RTN neurons in freely behaving mice and expanded in **(E)**. **(E)** Changes in RTN neurons adapting response to 3% CO_2_ (dotted box) with pre-hypercapnic baseline activity. **(F)** RTN neurons adapting response to 3% CO_2_ (from the dotted box in **(E)** measured as the area under the curve (AUC). Data in **(B, F)** are median and quartile and shown as continuous and dotted lines, respectively. For the comparison of E_A_ vs. non-E_A_ neurons in **(A)**, the chi-squared test is used with the false discovery rate procedure for the correction. One-way ANOVA with multiple comparisons and Brown–Forsythe correction are used for the comparison in **(B, F)**. F^*^ (DFn, DFd) are 51.64 (4.000, 110.4) for **(B)** and 4.869 (3.000, 21.21) for **(F)**. *P*-values are ^*^p < 0.05 and ^****^p < 0.0001.

Given that the adaptive ventilatory changes to hypercapnia altered following induction of SE, we tested whether there might also be changes in the activity of chemosensory neurons in the RTN. Previously, we have documented that the RTN neurons exhibit a range of firing patterns during hypercapnia (Bhandare et al., 2022), and in the present study, we also observed similar patterns of neuronal responses to hypercapnia from RTN neurons both before and after induction of SE (Figure 1B, Supplementary Figure 1). Our categorization of RTN neurons into the E_A_, E_G_, I, and NC subtypes was supported by a two-component analysis in which we plotted the change in Ca^2+^ activity (from baseline) elicited by 6% inspired CO_2_ vs. the change in Ca^2+^ activity elicited by 3% inspired CO_2_ (Figure 4 Aa-d x-y plots). As reported in a previous study (Bhandare et al., 2022), we found that the most frequent neuronal response to hypercapnia before induction of SE was an excited adapting (E_A_) pattern with an increase in activity immediately following the increase in inspired CO_2_ (Figures 1B, 4Aa donut chart).

Following induction of SE, changes in the pattern of RTN neuronal responses to hypercapnia became apparent over the following weeks (Figure 4). We performed the chi-squared test, corrected with the false discovery rate procedure for multiple comparisons (Curran-Everett, 2000), and found that the proportion of E_A_ neurons (vs. non-adapting subtypes) was significantly decreased at week 3 (week 3 vs. pre-SE, χ^2^ = 7.54, *p* = 0.006; week 5 vs. pre-SE, χ^2^ = 0.42, *p* = 0.51; week 7 vs. pre-SE, χ^2^ = 0.17, *p* = 0.68; Figures 1B, 4Aa-d donut charts). We noticed a similar trend when we looked at the baseline Ca^2+^ activity of E_A_ neurons (Figure 4B). Although the area under the curve (AUC) of the F/F_0_ in the baseline was significantly increased during SE compared to pre-SE (Figure 4B, *p* < 0.0001), at 3 weeks after SE this baseline activity was decreased compared to pre-SE level (Figure 4B, *p* = 0.03194). At later time points, the baseline activity recovered to pre-SE levels (Figure 4B). In contrast to the E_A_ neurons, we observed an increase in the proportion of E_G_ and I neurons after induction of SE in these mice with the greatest increase in the number of E_G_ neurons being evident at week 7 (Figure 4Aa-d donut charts). This suggests considerable plasticity in the chemosensory networks of the RTN.

Because E_A_ neurons were the major subtype in our recordings, we further quantified the adapting response by averaging the individual changes in GCaMP6 fluorescence at each monitoring point (Figures 4C–F). This calculation showed that the adapting Ca^2+^ signal was weaker at 3, 5, and 7 weeks following induction of SE compared to before SE (Figures 4D, E). As a further quantification, we measured the area under the curve (AUC) of the adapting response during 3% inspired CO_2_ (Figure 4F), which again confirmed the diminished neuronal activation during hypercapnia at weeks 5 and 7 post-SE (*p* = 0.016 and 0.011, respectively) (492.4 ± 109.0 at pre-SE compared to 337.4 ± 155.3, 155.6 ± 25.9 and 139.0 ± 24.9 at 3, 5, and 7 weeks post-SE, respectively). Overall, there was a transient reduction in the baseline E_A_ neuronal activity in the RTN at 3 weeks and also a sustained reduction in the amplitude of the responses of the E_A_ neurons to hypercapnia that was still evident 7 weeks post-SE induction.

### 3.5 Responses of the RVLM neurons to hypercapnia during chronic epilepsy

**Figure 5:**
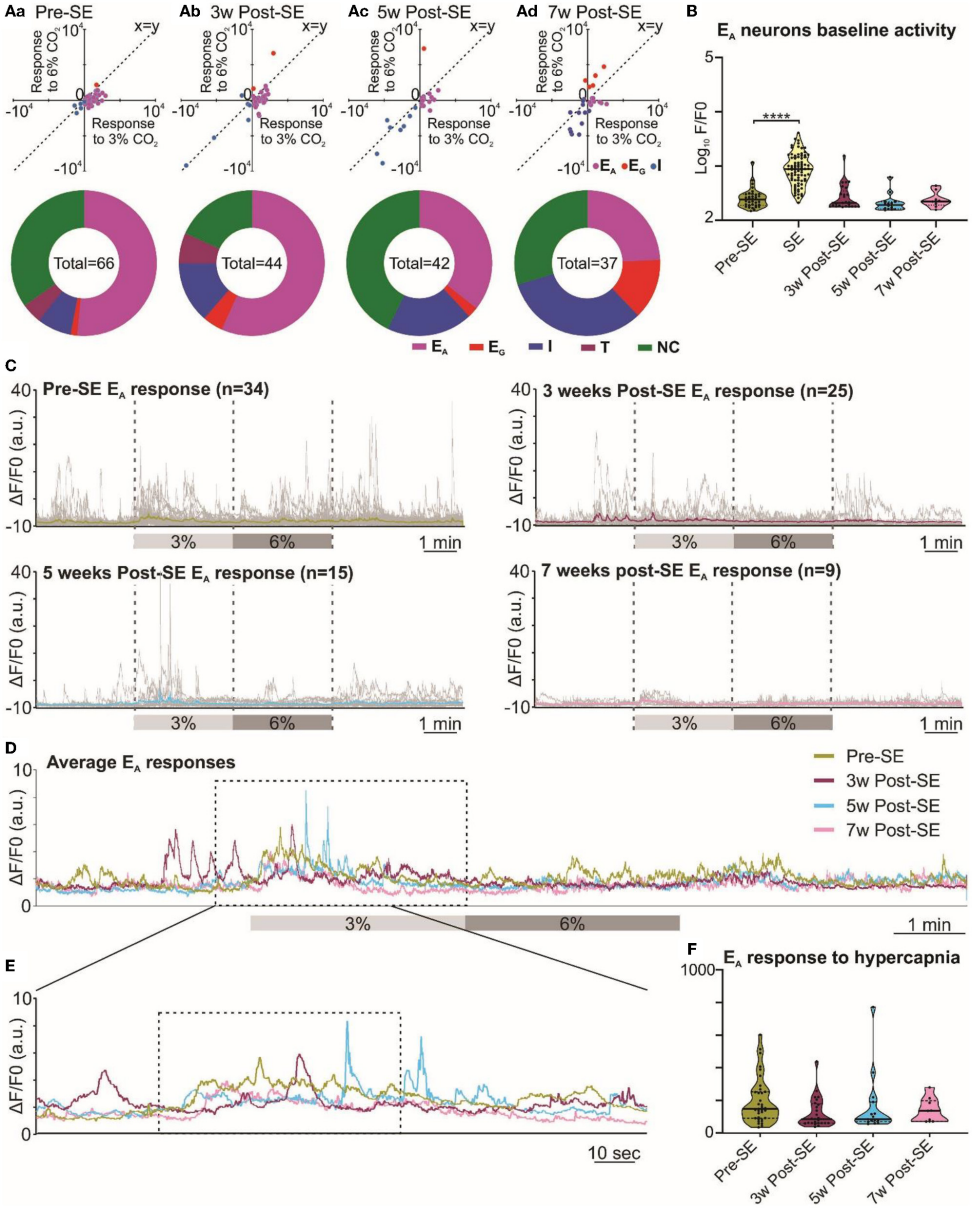
Responses of neurons in the RVLM are maintained following induction of SE. **(A)** Two-component analysis of neuronal categorization of RVLM cells at pre-SE (a) and 3 (b), 5 (c), and 7 weeks (d) after induction of SE. The change in Ca^2+^ signal (measured as the area under the curve) from baseline (room air) elicited by 6% CO_2_ plotted against the Ca^2+^ response elicited by 3% CO_2_. The line of identity (x = y) is shown. E_A_ neurons fall below this line, whereas E_G_ neurons fall above this line and I neurons are predominant in the negative quadrant of the graph. The donut charts represent the proportion of different types of neuronal responses to hypercapnia in the RVLM at pre-SE (a) and 3 (b), 5 (c), and 7 weeks (d) after induction of SE. **(B)** Baseline activities of RVLM E_A_ neurons at pre-SE, and 3, 5, and 7 weeks after induction of SE and compared with neuronal responses during SE. **(C)** RVLM neurons individual and average adapting responses to hypercapnia before and 3, 5, and 7 weeks after induction of epilepsy in freely behaving mice. n is the number of neurons recorded from 9 mice. **(D)** RVLM neurons average adapting responses compared between pre-SE and 3, 5, and 7 weeks after induction of SE. The dotted square shows changes in adapting response of RVLM neurons in freely behaving mice and expanded in **(E)**. **(E)** RVLM neurons adapting response to 3% CO_2_ (dotted box) with pre-hypercapnic baseline activity. **(F)** RVLM neurons adapting response to 3% CO_2_ (from the dotted box in **(E)** measured as the AUC. Data in the **(B, F)** are median and quartile and shown as continuous and dotted lines, respectively. For the comparison of E_A_ vs. non-E_A_ neurons in **(A)**, the chi-squared test is used with the false discovery rate procedure for the correction. One-way ANOVA with multiple comparisons and Brown–Forsythe correction are used for the comparison in **(B, F)**. F^*^ (DFn, DFd) are 81.34 (4.000, 113.7) for **(B)** and 1.651 (3.000, 38.93) for **(F)**. *P*-value is ^****^p < 0.0001.

Neurons of the RVLM group exhibited similar types of responses to hypercapnia as those in the RTN group (Figure 1B), and this was confirmed by a two-component analysis of the neuronal responses (Figure 5Aa-d x-y-plots). As for the RTN neurons, before induction of SE, the adapting response was most common (Figure 5Aa donut chart). Weeks after induction of SE, RVLM neuronal responses to hypercapnia showed a difference in the proportion of E_A_ neurons compared to non-adapting that was only evident at week 7 when tested with the chi-squared test and corrected with the false discovery rate procedure for multiple comparisons (week 3 vs. pre-SE χ^2^ = 0.3, *p* = 0.58; week 5 vs. pre-SE, χ^2^ = 2.58, *p* = 0.1; week 7 vs. pre-SE χ^2^ = 7.2, *p* = 0.007). There was also an increased proportion of inhibited and graded neurons at weeks 5 and 7 (Figure 5Aa-d donut charts). Baseline Ca^2+^ activity of RVLM adapting neurons (AUC of the F/F_0_) did not change during the development of chronic epilepsy, but, as expected, it showed a significant increase during induction of SE (*p* < 0.0001; Figure 5B). Averaging the GCaMP6 fluorescence activity of individual adapting RVLM neurons showed that these responses remained constant even after induction of SE (Figures 5C–F). However, the magnitudes of all RVLM responses (F/F_0_) were less than those of the RTN neurons (compare Figures 4C–F with Figures 5C–F). The AUC analysis of RVLM E_A_ neurons confirmed that their responses to hypercapnia remained stable at all time points after induction of SE (Figure 5F, AUC for RVLM adapting responses was 197.0 ± 24.0 at pre-SE compared to 123.2 ± 18.5, 163.2 ± 48.6, and 138.2 ± 24.8 at 3, 5, and 7 weeks post-SE, respectively).

### 3.6 Induction of SE and development of spontaneous seizures in the RTN and RVLM groups

**Figure 6:**
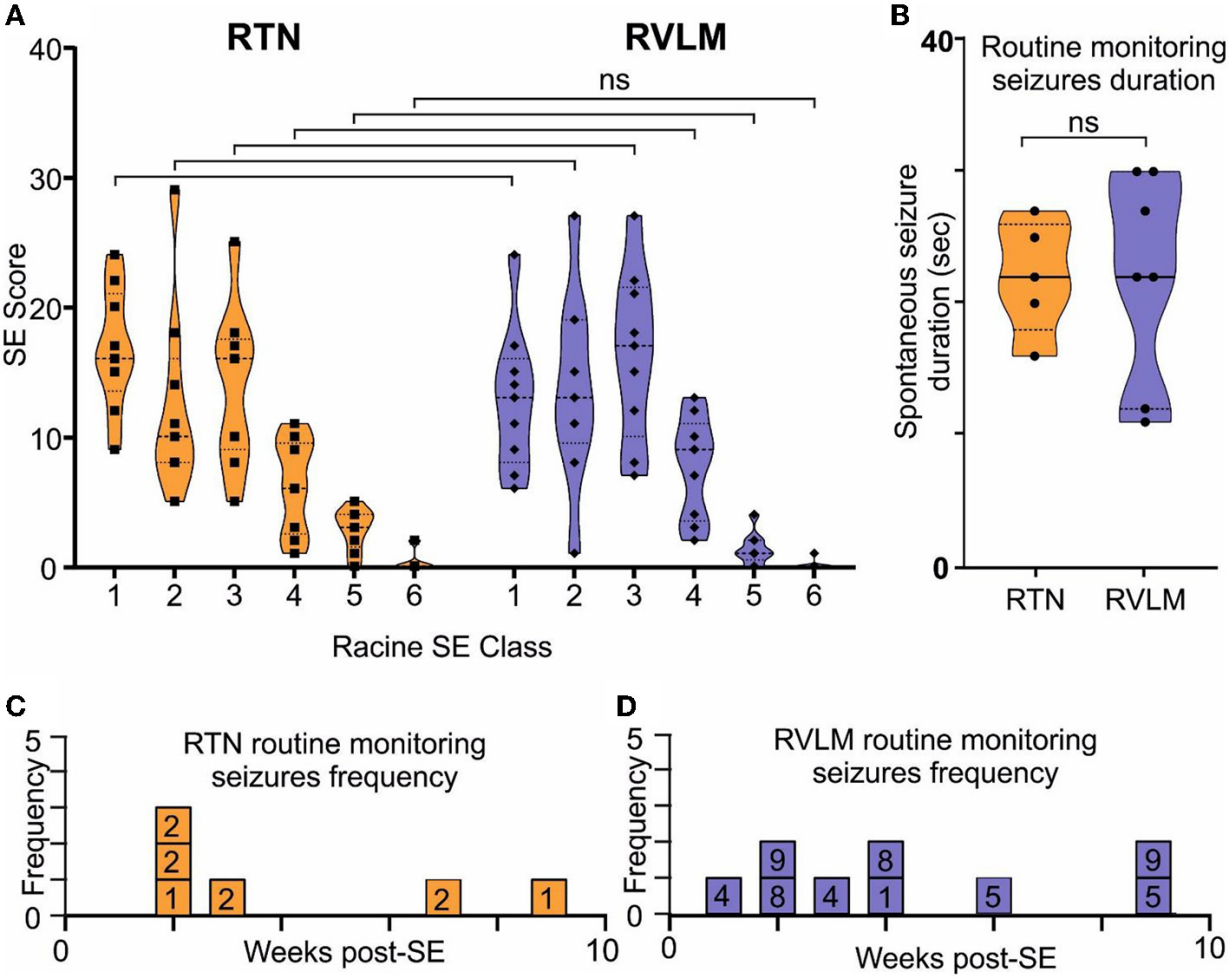
Comparable induction of SE and development of spontaneous seizures in the RTN and RVLM groups of mice. **(A)** Comparison of the Racine scale for behavioral scoring during induction of SE in mice recorded for RTN and RVLM. x-axis, Racine behavioral class, and y-axis, number (score) of specific SE behavior. **(B)** Comparison of duration of spontaneous seizures noted during routine monitoring and manually recorded in individual mice from the RTN and RVLM groups of mice. Weekly spontaneous seizure frequency noted (during routine mouse monitoring), and video recorded in individual mice recorded for **(C)** RTN and **(D)** RVLM neuronal activity after induction of SE. The numbers in the boxes in **(C, D)** are mouse identifiers and correspond to the identifying numbers in the Supplementary Figures 1–4. Data in **(A, B)** are median and quartile and are shown as continuous and dotted lines, respectively. For the comparison of the Racine scale score between the RTN and RVLM groups, the chi-squared test was used with the false discovery rate procedure for the correction. The Mann–Whitney non-parametric *t*-test was used for the comparison in **(B)**.

Chemoconvulsant-induced models of chronic epilepsy are variable with respect to the development of SE and the subsequent development of spontaneous seizures (Lévesque et al., 2016). Hence, to evaluate these parameters in both the RTN and RVLM groups of mice, we performed a series of quantitative analyses. First, we quantified the Racine behavioral scale values for each mouse and compared these values between the RTN and RVLM groups using the chi-squared test, which showed no difference between the two groups (Figure 6A; scale-1 χ^2^ = 0.29, *p* = 0.59; scale-2 χ^2^ = 0.05, *p* = 0.82; scale-3 χ^2^ = 0.11, *p* = 0.74; scale-4 χ^2^ = 0.14, *p* = 0.70; scale-5 χ^2^ = 1.77, *p* = 0.18; scale-6 χ^2^ = 1.43, *p* = 0.23).

**Figure 7:**
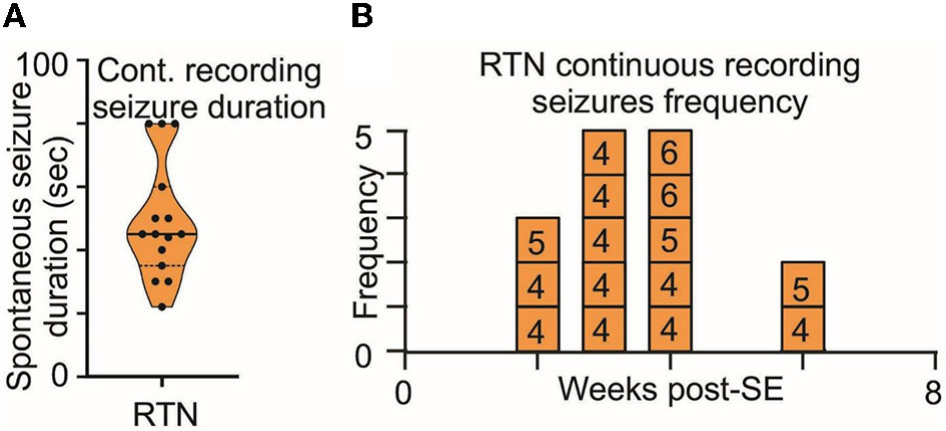
Long-term monitoring of spontaneous seizures in RTN mice. **(A)** Duration and **(B)** frequency of spontaneous seizures recorded in long-term video monitoring of three mice selected for recordings from the RTN neurons. Data in **(A)** are median and quartile and are shown as continuous and dotted lines, respectively. The numbers in the boxes in **(B)** are mouse identifiers and correspond to the identifying numbers in the Supplementary Figures 1–4.

Second, the duration and frequency of spontaneous seizures noted during routine animal monitoring were not different between RTN and RVLM (Figures 6B–D). To test whether the routine monitoring (30–45 min every day) was insufficient to capture the seizure behavior of the mice, we performed long-term continuous video monitoring in three additional RTN mice (6.9 h/day/mouse for 15 days over 7 weeks, Figures 7A–B). All three mice experienced spontaneous seizures with similar timing (between weeks 2 and 4 after SE) to the cohort of mice reported in Figure 6.

The routine monitoring was performed during the daily check-up of the mice over the 7 weeks post-SE and amassed approximately 30 h/mouse of visual inspection, which is approximately 3.5-fold shorter than the long-term video recording presented in Figure 7. However, as both methods gave results with similar numbers of seizures normalized to total recording time, we conclude that the routine daily monitoring was sufficient to sample the number, intensity, and duration of seizures exhibited by the mice.

## 4 Discussion

SUDEP is defined as a sudden, unexpected, witnessed or unwitnessed, non-traumatic, and non-drowning death occurring in benign circumstances in an individual with epilepsy, with or without evidence for a seizure and excluding documented status epilepticus (Ryvlin et al., 2013). One-third of people with epilepsy do not respond to currently available anti-epileptic medications and are more vulnerable to SUDEP. Furthermore, the incidence of SUDEP is higher in people with drug-resistant epilepsy (Tomson et al., 2008; Ryvlin et al., 2013). Case studies have found that poor seizure control, particularly of generalized tonic–clonic seizures (Walczak et al., 2001), a history of any seizure in the last 3 months before SUDEP (compared to none) (Langan et al., 2005), and increased frequency of tonic–clonic seizures per year (Nilsson et al., 1999) are significant risk factors for SUDEP.

Despite the correlative link between seizure history and SUDEP, its mechanism remains unclear. Although findings from human SUDEP cases and animal studies establish a central role for cardiac arrhythmia (Bateman et al., 2010; Bhandare et al., 2017), altered inter-ictal heart rate variability (Surges et al., 2009), cardiac arrest (Ryvlin et al., 2013), ventricular fibrillation (Naggar et al., 2014), bradycardia (Kalume et al., 2013), terminal apnoea (Ryvlin et al., 2013), obstructive respiratory apnoea (Jefferys et al., 2019; Irizarry et al., 2020), respiratory arrest (Faingold et al., 2010), and ictal/postictal hypoventilation (Bateman et al., 2010) as the proximal cause of SUDEP, the specific neuronal circuits involved, and the reasons for why their dysfunction leads to cessation of breathing or cardiovascular activity are unknown. This makes the prevention and treatment of SUDEP more difficult.

Modeling of SUDEP is experimentally challenging due to its unpredictable nature. While we have not directly modeled SUDEP, we microinjected a chemoconvulsant into a restricted brain region to induce localized acute and chronic spontaneous seizures. This approach allowed us to discern the effects of seizures on the central cardiorespiratory system that could contribute to SUDEP. In our study, chronic spontaneous seizures are defined as behavioral seizures as we did not record concurrent EEG activity in these mice. The chemoconvulsant-induced rodent models of epilepsy show a classical latent period after injection of chemoconvulsant, which is associated with reconfiguration of the neuronal network, neuronal loss, reactive gliosis, inflammation, and neurogenesis before the start of spontaneous seizures (Leite et al., 2002; Buckmaster, 2004). The latent period varies from model to model, but in intrahippocampal KA injection in mice, the latent period lasts, on average, between 4 and 30 days after KA administration (Levesque and Avoli, 2013; Lévesque et al., 2016; Rusina et al., 2021). In our study, mice were experiencing spontaneous seizures from week 2 after the injection of KA, and we noticed and video recorded these seizures during routine monitoring. Therefore, the neuronal and breathing changes that are reported at 3, 5, and 7 weeks after induction of SE are outside the classical latent period and are the effect of spontaneous seizures. More importantly, as mice were in the plethysmograph chamber for 30–60 min under observation before any recordings commenced, this excluded the possibility that the observed changes in neuronal activity and breathing were the effect of an immediately preceding seizure. However, we cannot exclude the possibility of seizures occurring a few hours or days before recording as we did not perform 24-h video recording to confirm this.

Adaptive increases in tidal volume (V_T_) or breathing frequency (f_R_) in response to hypercapnia (chemosensory reflex) are an important component of normal central respiratory homeostasis (Spyer and Gourine, 2009; Guyenet, 2014). Mice with chronic epilepsy developed a breathing phenotype with reduced V_T_ which was evident at 3 and 5 weeks after induction of SE. In chronic epileptic mice, baseline breathing in room air was reduced compared to the naive state, and their chemosensory adaptive responses to hypercapnia were significantly decreased at mild (3%) and moderate (6%) CO_2_. This reduced respiratory breathing phenotype in mice at weeks 3 and 5 after induction of SE has its neuronal correlate in the RTN adapting neuronal responses. Our findings are in line with a previous study where 30 days after induction of SE, rats showed a reduced breathing response (Maia et al., 2020). Similarly, people with epilepsy showed lower hypercapnic ventilatory responses correlated with a higher postictal rise in CO_2_ following generalized convulsive seizures (Sainju et al., 2019). Interestingly, at week 7 after induction of SE, we noticed a partial recovery of breathing phenotype in mice with chronic epilepsy possibly because of the emergence of neurons in the RTN that exhibited graded responses to hypercapnia. This suggests considerable adaptive plasticity in the respiratory neuronal networks, as has been shown in other contexts (Ling et al., 2001; Dahan et al., 2007).

By imaging neuronal Ca^2+^ activity at cellular resolution in key respiratory and cardiovascular brainstem nuclei in awake mice before, during, and after the establishment of the chronic epileptic state, we have avoided one of the major drawbacks of prior studies: the use of anesthesia which unavoidably alters circuit and cellular brain function. Clinical and preclinical findings have suggested that forebrain seizures might spread to the medullary neuronal network and cause central cardiorespiratory arrest (Aiba and Noebels, 2015; Dlouhy et al., 2015). Our real-time cellular imaging of the activity of individual neurons in the RTN and RVLM does indeed demonstrate that seizures spread into both nuclei and cause aberrant neuronal activity in these critical networks during and following the period of the seizure. This further strengthens the findings by Aiba and Noebels (2015) by establishing the effect of seizures on specific subsets of brainstem cardiorespiratory autonomic neurons. We have shown recently that the RTN neuronal responses do not change in mice due to their age or any other factor and, in fact, remain the same across weeks of interimaging interval (Figure 1–Figure Supplement 6 of Bhandare et al., 2022). Therefore, the change in the activity of brainstem neurons that we observed after induction of SE is the effect of seizures rather than an intrinsic time-dependent process in the brainstem.

The number of neurons recorded from session to session that we recorded from the same mouse varied, which is because movement artifacts (which may differ from session to session) sometimes prevent clear resolution of the activity of some neurons. We also encountered these issues in our previous study where we imaged neurons in the same region (Bhandare et al., 2022). The reasons for this are probably that the brainstem is extracranial and more mobile (hence the recordings are more susceptible to movement artifacts).

Our findings extend the observation of reduced hypercapnic ventilatory response in both animal models and people with epilepsy (Sainju et al., 2019) by providing further insights into (1) a potential underlying mechanism indicated by the alterations of neuronal responses in the RTN, a major chemosensory nucleus, and suggesting that (2) the central respiratory networks have sufficient plasticity to at least partially compensate for compromised breathing responses in epilepsy.

In addition to the ability of seizures to invade acutely into the RTN and RVLM nuclei, we found that the chronic epileptic state caused long-lasting alterations in the ability of RTN neurons to respond to hypercapnia. Adapting responses to hypercapnia evident in the naive state (before induction of SE) were greatly attenuated when tested 3, 5, and 7 weeks following establishment of the epileptic state (Figures 4C–F). This attenuation correlated with reduced hypercapnic and normal room air breathing responses at weeks 3 and 5 post-SE in these mice. On the other hand, the proportion of excitatory graded RTN neurons increased at week 7 post-SE, which correlates with the evidence of partial recovery of hypercapnic breathing response at 7 weeks in these mice.

The seizure delay time (time to induce first electrographic seizure after KA injection) was decreased during chronic epilepsy in both RTN and RVLM mice (compared to the delay time for the initial SE seizures) when we injected a 6-fold lower dose of KA. This decrease suggests the faster propensity of seizure invention into the brainstem during chronic epilepsy and an increased risk of cardiorespiratory brainstem circuit impairment. Importantly, seizure duration and delay time do not differ between the RTN and RVLM groups of mice during both SE and chronic seizures.

Analysis of neuronal Ca^2+^ activity during induction of SE showed that the average activity of the RTN and RVLM neurons was increased to a similar level suggesting that both nuclei had a similar effect of induction of SE. Additionally, when we compared all neuronal activities after injection of KA during induction of SE and subthreshold KA-induced seizures, these activities were also not different between RTN and RVLM mice (Figures 2H, I). This result suggests that both the RTN and RVLM mice had an equal SE trigger and development of comparable spontaneous seizures. Therefore, the lack of effect of spontaneous seizures on the responses of RVLM adapting neurons to hypercapnia is unlikely to be because the SE or spontaneous seizures were weaker in these mice.

Our longitudinal imaging of neurons before and weeks after induction of SE enabled us to document the effects of repeated chronic spontaneous seizures, a major risk factor for SUDEP, on the function of cardiorespiratory neurons and hypercapnic breathing responses. The weakening of the chemosensory reflex via loss of chemosensitivity in the RTN could plausibly increase the risk of SUDEP. For example, we speculate that if breathing was to cease due to invasion of spreading depolarisation and silencing of electrical activity into the brainstem, arterial blood would become hypercapnic and acidic. Ordinarily, this would act as a powerful stimulant to restart breathing once the spreading depolarisation had passed. However, the weakened chemosensory responses of RTN neurons that we observed in the chronic epileptic state might make it harder to restart breathing and could thus increase the risk of SUDEP. This speculation needs to be tested in a more specific animal model of SUDEP, e.g., DBA/1 mice where the incidence of respiratory arrest following audiogenic seizures increases with seizure repetition (Faingold et al., 2010) and DBA/2, where mice have a 100% incidence of respiratory arrest depending on their age. Although these mice die from obstructive apnoea, these deaths are likely to arise from reduced muscle tone and respiratory-related central drive that serves to dilate the airways during inspiration (Irizarry et al., 2020).

KA-induced SE and subsequent spontaneous seizures in mice facilitate a systematic understanding of changes in neuronal and breathing responses to hypercapnia before and after the induction of epilepsy. However, KA-induced epilepsy in mice is not a model of SUDEP and does not replicate all other pathological features associated with specific subclasses of epilepsy such as congenital genetic anomalies, sodium, potassium, serotonin, and calcium channelopathies. An alternative chemoconvulsant, pilocarpine, -induced SE is also a widely used model of temporal lobe epilepsy. Both KA- and pilocarpine-induced models of epilepsy have their own pros and cons, which have been extensively discussed previously (Covolan and Mello, 2000) and need to be considered during study design. Despite these differences, KA-induced SE produces neuropathological changes that are similar to those occurring in some patients with epilepsy (Lévesque et al., 2016).

While we have not studied the effect of seizures on chemosensory neurons of the raphe nucleus, agonists for serotonin receptors (a key raphe neurotransmitter) have a protective effect in blocking seizure-induced SUDEP in DBA mice (Faingold et al., 2011; Ma et al., 2022) and mice with genetic deletion of serotonin neurons (Buchanan et al., 2014). Raphe neurons project to the RTN (Wu et al., 2019) and the pre-Bötzinger complex (Ma et al., 2022) and contribute to chemosensory responses in these nuclei. Thus, activation of serotonin receptors could help to compensate for the weakened responses of the chemosensitive RTN neurons and enhance the stimulatory effect of CO_2_ on breathing (Mulkey et al., 2007; Wu et al., 2019). This could also be the likely mechanism of partial recovery of breathing responses at week 7 post-SE and the emergence of graded response in the RTN. Nevertheless, the action of serotonin could also be mediated through its direct effect on seizure activity (Buchanan et al., 2014; Schoonjans et al., 2017), arousal (Buchanan and Richerson, 2010), sleep-wake regulation, and circadian rhythm (Miyamoto et al., 2012) and warrants further investigation.

In contrast to altered RTN neuronal responses to hypercapnia during chronic epilepsy, RVLM neurons showed no change in their hypercapnic responses after SE. Findings from epilepsy monitoring units (So et al., 2000; Bateman et al., 2010; Ryvlin et al., 2013; Mueller et al., 2014; Dlouhy et al., 2015) and preclinical studies (Faingold et al., 2010; Jefferys et al., 2019; Maia et al., 2020) indicate that respiratory arrest rather than cardiac failure is the major contributor to SUDEP. Our findings are thus consistent with this result as they show a greater immediate effect of repeated seizures on the RTN chemosensory neurons than on the RVLM neurons. Although either central respiratory or obstructive apnoea contributes to SUDEP, it sets in train a sequence of events that include hypoxemia and acidosis that could indirectly trigger the failure of the central cardiovascular system and eventually cardiac arrest (Ryvlin et al., 2013).

A final implication of our findings is that longitudinal monitoring of respiratory chemosensitivity, combined with other established clinical risk factors, might be a useful biomarker test to identify people at risk of SUDEP. Although a pre-epileptic baseline for respiratory chemosensitivity could not be established in people with epilepsy, regular measurement of chemosensitivity via a simple non-invasive breathing test, which can be readily performed in epilepsy monitoring units (Sainju et al., 2019), could establish trends in chemo-responsiveness to indicate increased or diminished risk of SUDEP.

## Data availability statement

The original contributions presented in the study are included in the article/Supplementary material, further inquiries can be directed to the corresponding author.

## Ethics statement

Experiments were performed in accordance with the European Commission Directive 2010/63/EU (European Convention for the Protection of Vertebrate Animals used for Experimental and Other Scientific Purposes) and the United Kingdom Home Office (Scientific Procedures) Act (1986) with project approval from the University of Warwick's AWERB (PP1674884). The study was conducted in accordance with the local legislation and institutional requirements.

## Author contributions

AB: Conceptualization, Data curation, Formal analysis, Funding acquisition, Investigation, Methodology, Project administration, Resources, Software, Validation, Visualization, Writing – original draft, Writing – review & editing. ND: Conceptualization, Formal analysis, Funding acquisition, Project administration, Supervision, Writing – review & editing.
