# Supplementary material for: Neural correlate of reduced respiratory chemosensitivity during chronic epilepsy
Source: Front Cell Neurosci. 2023 Dec 20;17:1288600. doi: 10.3389/fncel.2023.1288600 (PMC10773801; doi:10.3389/fncel.2023.1288600)
Supplement: Supplementary file 5 [file Data_Sheet_1.DOCX]

Neural correlate of reduced respiratory chemosensitivity during chronic epilepsy

Supplementary Material

## Supplementary Figures


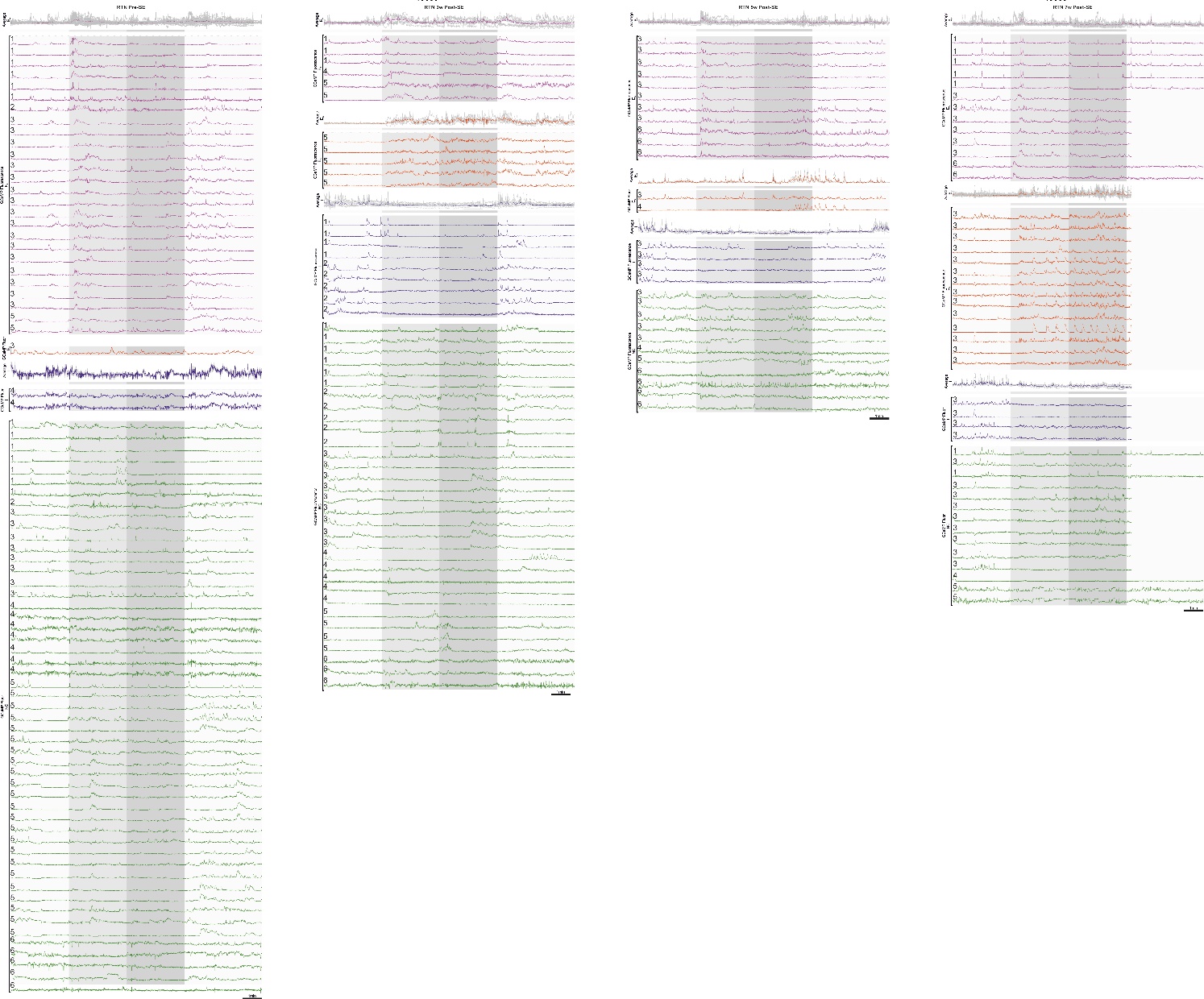


**Supplementary Figure 1:** **RTN neurons’ responses to the hypercapnic challenge at different time points in epilepsy.** RTN excited graded (E_G_), excited adapting (E_A_), inhibited (I), and non-coding (NC) neuronal responses, at pre-SE, and 3-, 5- and 7-weeks after induction of SE, time-matched with hypercapnia (light grey-3% CO_2_, medium grey-6% CO_2_) and average waveforms of E_G_, E_A_, and I. Animal number is indicated on the left hand side of the neuronal trace. In week-7 post-SE RTN neurons, for technical reasons the recording of recovery from hypercapnia is absent.


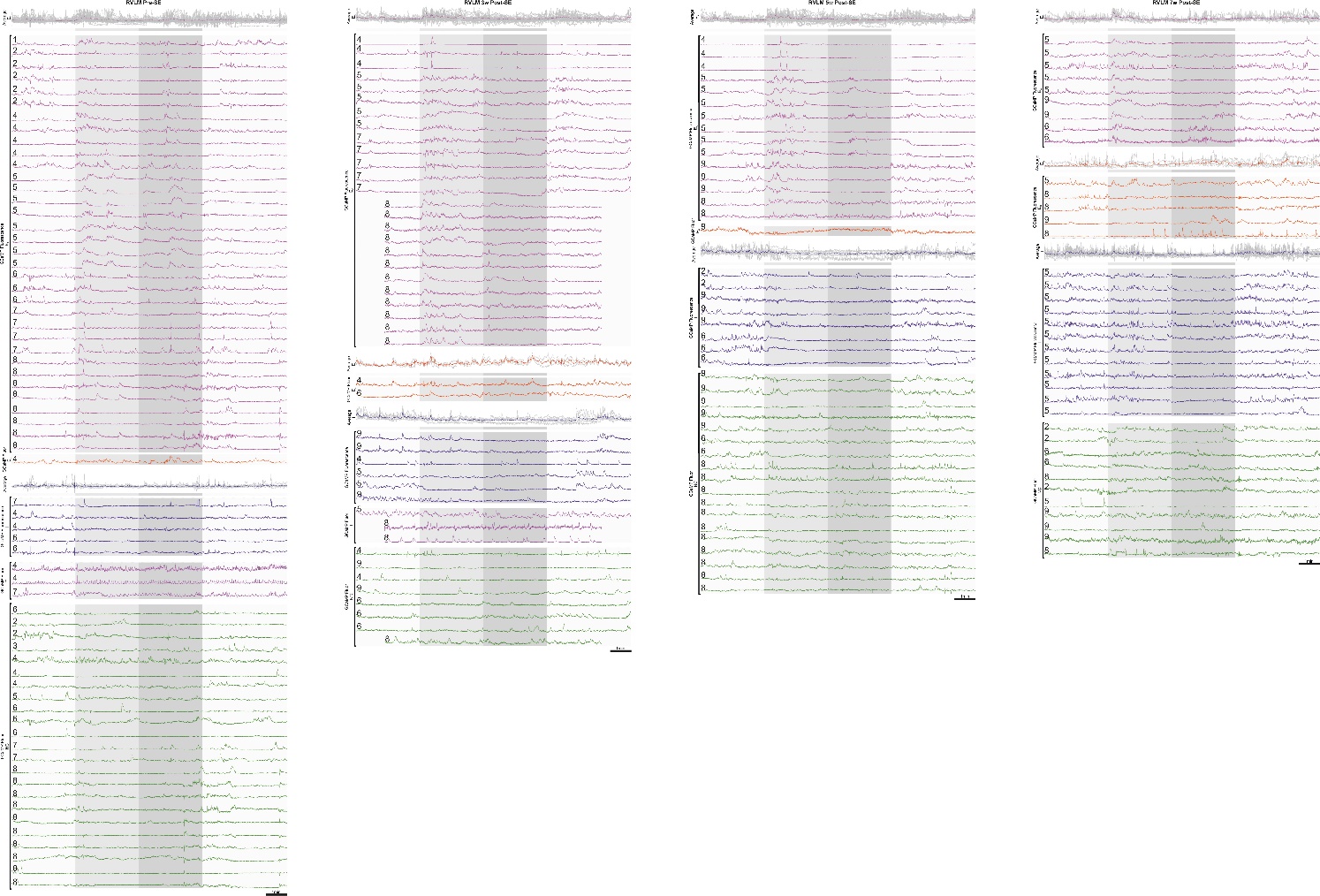


**Supplementary Figure 2:** **RVLM neurons’ responses to the hypercapnic challenge at different time points in epilepsy.** RVLM excited graded (E_G_), excited adapting (E_A_), inhibited (I), tonic (T), and non-coding (NC) neuronal responses, at pre-SE, and 3-, 5- and 7-weeks post-SE, time-matched with hypercapnia (light grey-3% CO_2_, medium grey-6% CO_2_) and average waveforms of E_G_, E_A_, and I. Animal number is indicated on the left hand side of the neuronal trace.


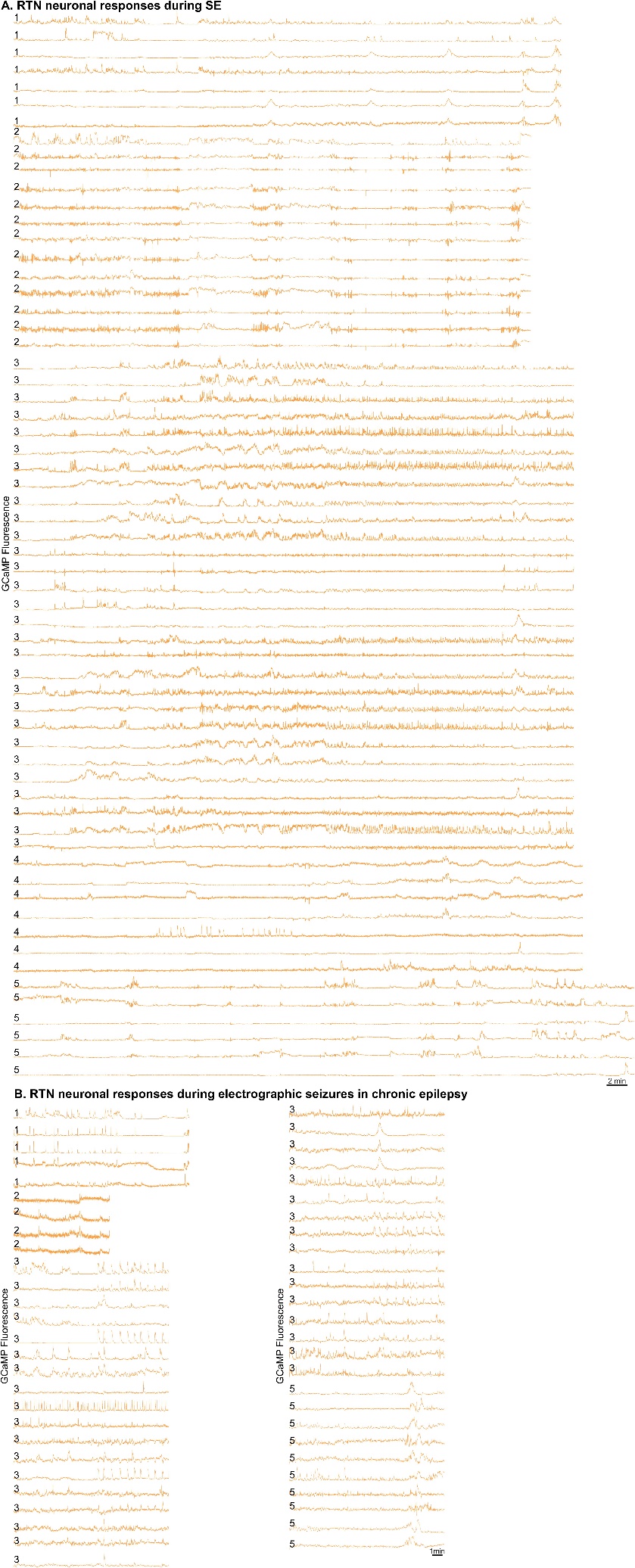


**Supplementary Figure 3:** **RTN neurons’ activity during KA induced SE and subthreshold KA-induced seizures during chronic epilepsy.** Animal number is indicated on the left hand side of the neuronal trace.


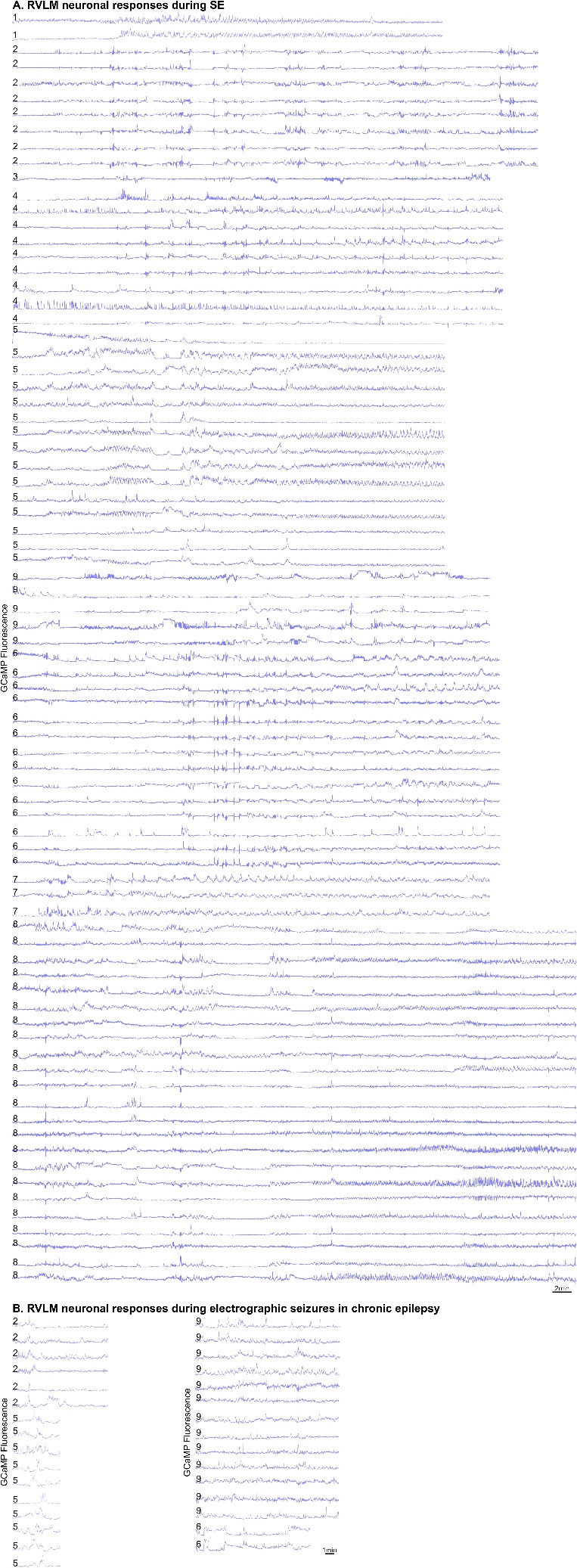


**Supplementary Figure 4:** **RVLM neurons’ activity during KA induced SE and subthreshold KA-induced seizures during chronic epilepsy.** Animal number is mentioned on the left hand side of the neuronal trace.


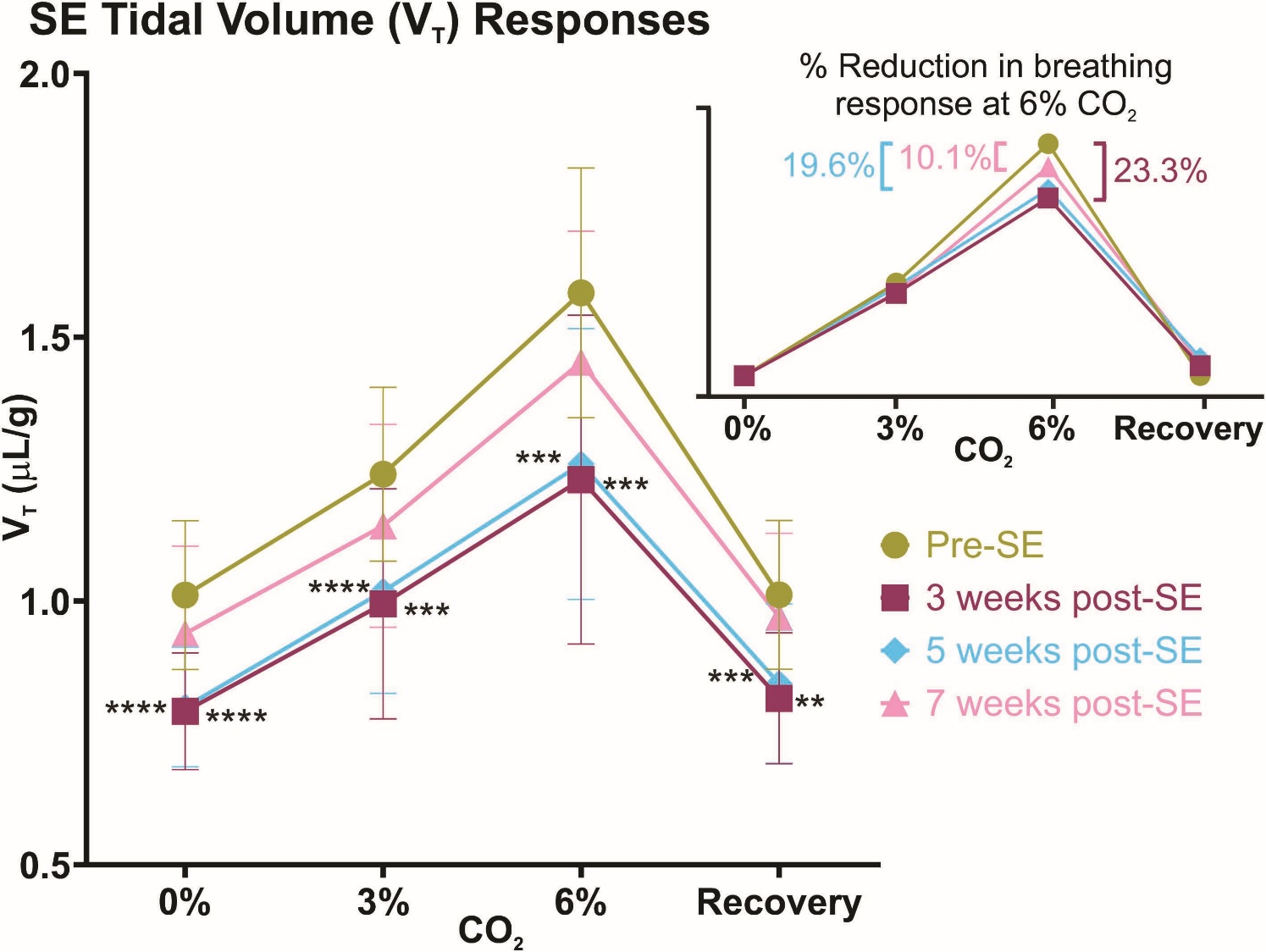


**Supplementary Figure 5. Replot of the data in Figure 3 as V_T_ (tidal volume) versus percent inspired CO_2_ pre-SE and at 3, 5 and 7 weeks after induction of SE.** Data presented as mean ± SD. Note that V_T_ is suppressed at all levels of CO_2_ at 3 and 5 weeks after induction of SE, but recovers by 7 weeks post-SE. Figure inset shows % decrease in chemosensitivity in response to 6% CO_2_ challenge at 3, 5 and 7 weeks after induction of SE compared to pre-SE level in the same mice. P values derived from two-way repeated measure (mixed effects) ANOVA with Tukey’s multiple comparison are **p* < 0.05, ***p* < 0.001, ****p* < 0.001 and *****p* < 0.0001.

# Supplementary Movies

**Supplementary Movie 1:** Seizures spread into the RTN neurons and disturb their activity during induction of SE via intrahippocampal KA injection in freely behaving mice. Movie is 8x fast forwarded. Selective neuronal traces are shown and matched with the ROIs drawn around GCaMP6s fluorescent neuronal cell bodies.

**Supplementary Movie 2:** Seizures spread into the RVLM neurons and disturb their activity during induction of SE via intrahippocampal KA injection in freely behaving mice. Movie is 8x fast forwarded. Selective neuronal traces are shown and matched with the ROIs drawn around GCaMP6s fluorescent neuronal cell bodies.

**Supplementary Movie 3:** During chronic epilepsy, 3 times of lower dose of KA (0.1 μg) compared to SE showed reduced latency for induction and spread of seizures into the RVLM neurons. Movie is 8x fast forwarded. Selective neuronal traces are shown and matched with the ROIs drawn around GCaMP6s fluorescent neuronal cell bodies.

**Supplementary Movie 4:** During chronic epilepsy, 6 times of lower dose of KA (0.05 μg) compared to SE showed reduced latency for induction and spread of seizures into the RTN neurons. Movie is 8x fast forwarded. Selective neuronal traces are shown and matched with the ROIs drawn around GCaMP6s fluorescent neuronal cell bodies.
